# Redefining CHI3L1: Therapeutic Opportunities at the Crossroads of Immune Suppression and Disease Progression

**DOI:** 10.1002/med.70076

**Published:** 2026-07-16

**Authors:** Kirti Upmanyu, Saurabh Upadhyay, Moustafa T. Gabr

**Affiliations:** ^1^ Department of Radiology, Molecular Imaging Innovations Institute (MI3) Weill Cornell Medicine New York New York USA

**Keywords:** CHI3L1, fibrosis, IL‐13Rα2–TMEM219–Galectin‐3 receptor triad, immune resistance, immunometabolism, pseudoenzymatic immune checkpoint, structural glycobiology, translational therapeutics, YKL‐40

## Abstract

Chitinase‐3‐like‐1 (CHI3L1, also known as YKL‐40) has been recognized as a biomarker of inflammation and tissue remodeling and has now emerged as a pseudoenzymatic immune checkpoint. Recent structural, immunological, and translational studies redefine it as an active regulator of immune suppression rather than a passive disease marker. Despite lacking catalytic activity, CHI3L1's conserved chitinase fold and glycan‐decorated surface acts as a modular scaffold linking cytokine, metabolic, and stress signals to immune suppression and fibrotic remodeling. Through its chitinase‐like structure it engages with receptor triad complex, including IL‐13Rα2, TMEM219, and Galectin‐3, activating signaling pathways such as MAPK, PI3K/AKT, and TGF‐β/Smad. Through these interactions, CHI3L1 has been reported to promote fibroblast activation, angiogenesis, and immune suppression, creating environments where immune cells are excluded or silenced. These checkpoint‐like effects are not limited to cancer; they also appear in fibrosis, infection, and neuroinflammatory diseases, linking CHI3L1 to a broad spectrum of immune‐resistant conditions. This review integrates structural glycobiology, receptor pharmacology, and immunometabolic mechanisms to reveal CHI3L1 as a unifying regulator of stromal–immune crosstalk. It further highlights preclinical and clinical evidence demonstrating that antibody, RNA‐based, and small‐molecule inhibitors of CHI3L1 restore immune surveillance and limit pathological remodeling. Targeting this pseudoenzymatic checkpoint alongside canonical PD‐1 and CTLA‐4 blockade represents a promising strategy to overcome immune resistance and normalize pathogenic tissue remodeling across diverse diseases.

## Summary

- CHI3L1 is redefined as a pseudoenzymatic immune checkpoint that integrates structural, metabolic, and immunologic control.
- The IL‐13Rα2–TMEM219–Galectin‐3 receptor triad mediates CHI3L1‐driven MAPK, PI3K/AKT, and TGF‐β/Smad signaling.
- CHI3L1 coordinates fibroblast activation, macrophage polarization, and endothelial remodeling to enforce immune exclusion.
- Antibody, small‐molecule, and RNA‐based inhibitors restore T‐cell activation and suppress fibrosis in preclinical models.
- Targeting CHI3L1 alongside PD‐1/CTLA‐4 checkpoints offers a new therapeutic avenue against immune‐resistant diseases.

**Abbreviations**
AD: Alzheimer's disease
AKT: protein kinase B
ASO: antisense oligonucleotide
Aβ: amyloid‐β
BACE1: β‐site amyloid precursor protein–cleaving enzyme 1
CAP: community‐acquired pneumonia
CD: cluster of differentiation
CHI3L1: chitinase‐3‐like protein 1 (YKL‐40)
CNS: central nervous system
COPD: chronic obstructive pulmonary disease
CRTH2: chemoattractant receptor‐homologous molecule expressed on Th2 cells
CTLA‐4: cytotoxic T‐lymphocyte–associated protein 4
ECM: extracellular matrix
ERK: extracellular signal‐regulated kinase
FVC: forced vital capacity
HIF‐1α: hypoxia‐inducible factor‐1 alpha
HR: hazard ratio
IBD: inflammatory bowel disease
ICB: immune checkpoint blockade
ICOS: inducible T‐cell co‐stimulator
IFN‐γ: interferon‐gamma
IKKβ: IκB kinase beta
IL: interleukin
IPF: idiopathic pulmonary fibrosis
iPSC: induced pluripotent stem cell
JNK: c‐Jun N‐terminal kinase
KL‐6: Krebs von den Lungen‐6 (MUC1)
LAG‐3: lymphocyte activation gene‐3
MAPK: mitogen‐activated protein kinase
MMP: matrix metalloproteinase
MS: multiple sclerosis
NET: neutrophil extracellular trap
NF‐κB: nuclear factor kappa B
NK: natural killer
NSCLC: non‐small cell lung cancer
PD‐1: programmed cell death protein 1
PD‐L1: programmed death‐ligand 1
PET: positron emission tomography
PI3K: phosphoinositide 3‐kinase
PTEN: phosphatase and tensin homolog
RA: rheumatoid arthritis
RLH: RIG‐I–like helicase
RNAi: RNA interference
RSV: respiratory syncytial virus
siRNA: small interfering RNA
SIRPα: signal regulatory protein alpha
SMAD: mothers against decapentaplegic homolog
SOFA: sequential organ failure assessment
STAT3: signal transducer and activator of transcription 3
TAM: tumor‐associated macrophage
TBI: traumatic brain injury
TGF‐β: transforming growth factor beta
Th2: T helper type 2
TME: tumor microenvironment
TMEM219: transmembrane protein 219
TNBC: triple‐negative breast cancer
TNF‐α: tumor necrosis factor alpha
Treg: regulatory T cell
VEGF: vascular endothelial growth factor
VOC: variant of concern
YAP/TAZ: yes‐associated protein/transcriptional coactivator with PDZ‐binding motif.

## 1 Introduction

Chitinase‐like proteins (CLPs) occupy a paradoxical niche within the glycoside‐hydrolase 18 (GH18) family as they retain the structural chitinase fold but lack catalytic activity [1]. Among them, chitinase‐3‐like 1 (CHI3L1, also known as YKL‐40 in humans) has emerged as a pseudoenzymatic regulator that retains structural conservation while acquiring immunomodulatory function. Initially identified in MG‐63 osteosarcoma cells, CHI3L1 is now known to be expressed in chondrocytes, synoviocytes, vascular smooth‐muscle cells, immune and stromal cells, hepatocytes, endothelial cells, and diverse tumors [2]. Although catalytically inactive, its conserved structural scaffold endows CHI3L1 with pseudoenzymatic immune checkpoint‐like activity, enabling the regulation of tissue remodeling, angiogenesis, and immune suppression [3]. Functionally, CHI3L1 integrates growth, survival, and immune‐signaling pathways by promoting proliferation of chondrocytes, fibroblasts, and epithelial cells through MAPK and PI3K/AKT signaling, and protecting cardiomyocytes and cancer cells from apoptosis via Akt activation [4]. Immunologically, it has been shown to promote macrophage maturation, dendritic‐cell recruitment, and Th2 polarization [5], while remodeling the extracellular matrix (ECM) through regulation of MMP activity and E‐cadherin expression to foster fibrosis and invasion [6]. These emerging findings now redefine CHI3L1 not just as a passive biomarker but as an active pseudoenzymatic immune checkpoint, mediating signaling functions that suppress immune activation, sustain remodeling programs, and promote immune resistance across pathological contexts.

At the genetic level, CHI3L1 expression is regulated by alternative transcripts and promoter polymorphisms that influence circulating protein levels [7]. Genome‐wide association studies identify certain CHI3L1 alleles as active determinants of disease susceptibility, linking specific SNPs to asthma, inflammatory bowel disease, and neurodegeneration [8, 9]. Despite these divergent outcomes, CHI3L1 consistently remodels the tissue–immune interface: in cancer, it drives immune exclusion and angiogenesis; in fibrosis, persistent fibroblast activation and matrix deposition; in chronic inflammation, macrophage skewing and stromal activation; and in neurodegeneration, astrocyte and microglial reprogramming [10, 11]. CHI3L1 not only marks these processes but actively coordinates them by modulating macrophage polarization, dampening inflammation, and promoting angiogenesis and remodeling, it drives macrophages toward repair, fibroblasts toward fibrosis, and glia toward maladaptive remodeling, converging on immune resistance [12, 13].

To clearly define the conceptual framework used in this review, immune checkpoints are considered here based on functional criteria rather than strictly structural features. Canonical checkpoints such as PD‐1 and CTLA‐4 regulate T‐cell activation through receptor–ligand interactions at the immune synapse [14]. More broadly, immune checkpoints are defined by their ability to impose dominant inhibitory control over immune activation, particularly under conditions of chronic inflammation, tissue stress, or persistent antigen exposure [15]. Key functional attributes include suppression of co‐stimulatory signaling, induction of inhibitory pathways, establishment of negative feedback loops, and restoration of immune activity upon blockade. These criteria are increasingly used to describe both synaptic and non‐synaptic regulators of immune suppression.

In this context, CHI3L1 represents a mechanistically distinct but functionally convergent regulator. As a secreted glycoprotein, it does not directly engage T‐cell receptors but instead enforces immune suppression indirectly by remodeling stromal, metabolic, and vascular components of the tissue microenvironment [12]. Mechanistically, CHI3L1 has been reported to suppress co‐stimulatory pathways such as CD28 and ICOS signaling, has been also reported to induce inhibitory pathways including PD‐L1 expression, and has been shown to promote macrophage, fibroblast, and endothelial reprogramming that collectively establish immune‐excluded niches [6, 16, 17]. We therefore define CHI3L1 as a “pseudoenzymatic immune checkpoint,” a non‐canonical regulator reflecting both its lack of catalytic activity and its extracellular, microenvironment‐driven mode of immune regulation, which distinguishes it from canonical synaptic checkpoints while preserving core functional properties.

Consistent with these functional criteria, CHI3L1 exhibits the hallmarks of an immune checkpoint [11, 17, 18]. Unlike canonical checkpoints such as PD‐1 and CTLA‐4, which regulate T‐cell activation through receptor–ligand interactions at the immune synapse, CHI3L1 has been reported to function as a secreted extracellular pseudoenzymatic checkpoint that enforces immune suppression in trans by remodeling the surrounding microenvironment rather than engaging immune receptors directly. Its secretion allows it to act systemically and spatially apart from cell–cell interfaces, integrating stromal, immune, and vascular cues to create an immunosuppressive niche. It inhibits T‐cell co‐stimulation, interferes with ICOS–ICOSL and CD28 (Figure 1) phosphorylation, has been reported to induce STAT3‐driven PD‐L1 expression, and shapes tumor and fibrotic microenvironments toward immune exclusion [16, 17]. By integrating extracellular, structural, and metabolic cues, CHI3L1 orchestrates coordinated suppressive outcomes. Clinically, resistance to PD‐1 blockade, limited antifibrotic progress, and the lack of immunomodulatory options in neurodegeneration all implicate CHI3L1 as a hidden bottleneck in immune regulation [10, 17].

**Figure 1:**
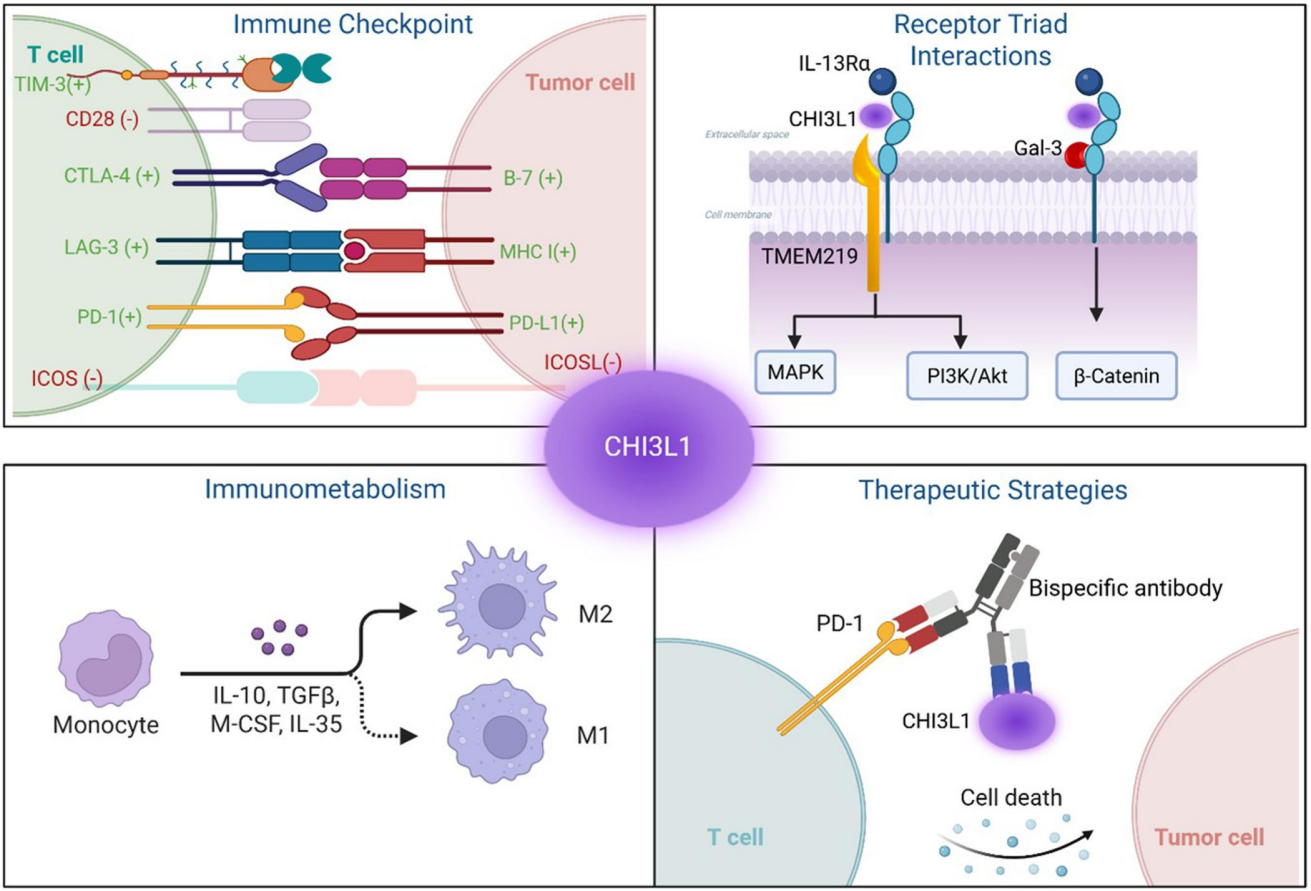
Multifaceted roles of CHI3L1 in immune regulation and therapy. CHI3L1 acts as a non‐canonical immune checkpoint that suppresses T‐cell co‐stimulation and enhances inhibitory pathways (PD‐1/PD‐L1, CTLA‐4/B7). It signals through the IL‐13Rα2–TMEM219–Galectin‐3 triad to activate MAPK, PI3K/AKT, and β‐catenin pathways, reprograms macrophages toward an M2 phenotype, and serves as a therapeutic target—where bispecific PD‐1 × CHI3L1 antibodies restore T‐cell cytotoxicity and promote tumor cell death. [Color figure can be viewed at wileyonlinelibrary.com]

Reframing CHI3L1 as an immune checkpoint unifies diverse pathologies under a common therapeutic logic. It connects CHI3L1 to immuno‐oncology (Figure 1) efforts aimed at overcoming resistance to PD‐1 and CTLA‐4 blockade, extends checkpoint principles to fibrotic and neuroinflammatory diseases, and identifies CHI3L1 as both biomarker and drug target [16]. Inhibiting CHI3L1 could complement existing checkpoint, antifibrotic, and anti‐angiogenic therapies, transforming its broad biological influence into a unified therapeutic opportunity [19].

This review integrates the structural, molecular, and immunological dimensions of CHI3L1. It highlights its checkpoint‐like properties, details receptor‐interface pharmacology via the IL‐13Rα2–TMEM219–Galectin‐3 triad (Figure 1), explores its roles in immunometabolism across macrophages, fibroblasts, and endothelial cells, and outlines translational opportunities from drug discovery to biomarker‐guided clinical validation [20]. The discussion then highlights CHI3L1's influence on immunometabolism, illustrating how it reprograms macrophages, fibroblasts, and endothelial cells to enforce tissue‐level immune resistance. Finally, the review charts a translational roadmap, encompassing drug development, biomarker strategies, and clinical validation. Together, these insights redefine CHI3L1—not merely as a disease‐associated marker but as a targetable checkpoint orchestrating immunity across cancer, fibrosis, chronic inflammation, and neurodegeneration.

## 2 Structure and Molecular Features of CHI3L1

CHI3L1 is encoded on chromosome 1q32.1 and composed of 383 amino acids with a molecular weight of approximately 40 kDa. The protein lost its chitinase activity due to a single amino‐acid substitution within the conserved DXDXE motif, where glutamic acid is replaced by leucine [12]. Rather than a loss, this mutation converted CHI3L1 into a pseudoenzymatic scaffold that regulates immunity, fibrosis, and cancer. The first X‐ray structure of CHI3L1 (PDB 1HJW, 2.6 Å) revealed a two‐domain architecture centered on a (β/α)_8_ TIM barrel featuring a broad chitin‐binding cleft along the C‐terminal ends of its β‐strands [21]. The second domain comprises six antiparallel β‐strands and a single α‐helix inserted after β7 of the (β/α)_8_ barrel, forming a compact bispherical fold. Co‐crystal structures with chitin fragments [(GlcNAc)_2_–(GlcNAc)\_5_] mapped a 43 Å carbohydrate‐binding groove containing nine subsites (–6 to +3) at the C‐terminal ends of the β‐strands [22]. This groove is lined with aromatic residues such as Trp99 and Trp352 that mediate hydrophobic stacking and hydrogen bonding for ligand recognition, while residues Arg263 and Glu70 provide additional specificity for N‐acetylglucosamine. Although catalytically inactive, this pseudoactive groove retains glycan‐binding capacity and conformational flexibility, enabling CHI3L1 to engage multiple receptors and influence tissue remodeling, angiogenesis, and immune suppression [23]. These structural features, including the conserved TIM‐barrel fold and carbohydrate‐binding groove, are illustrated in Figure 2.

**Figure 2:**
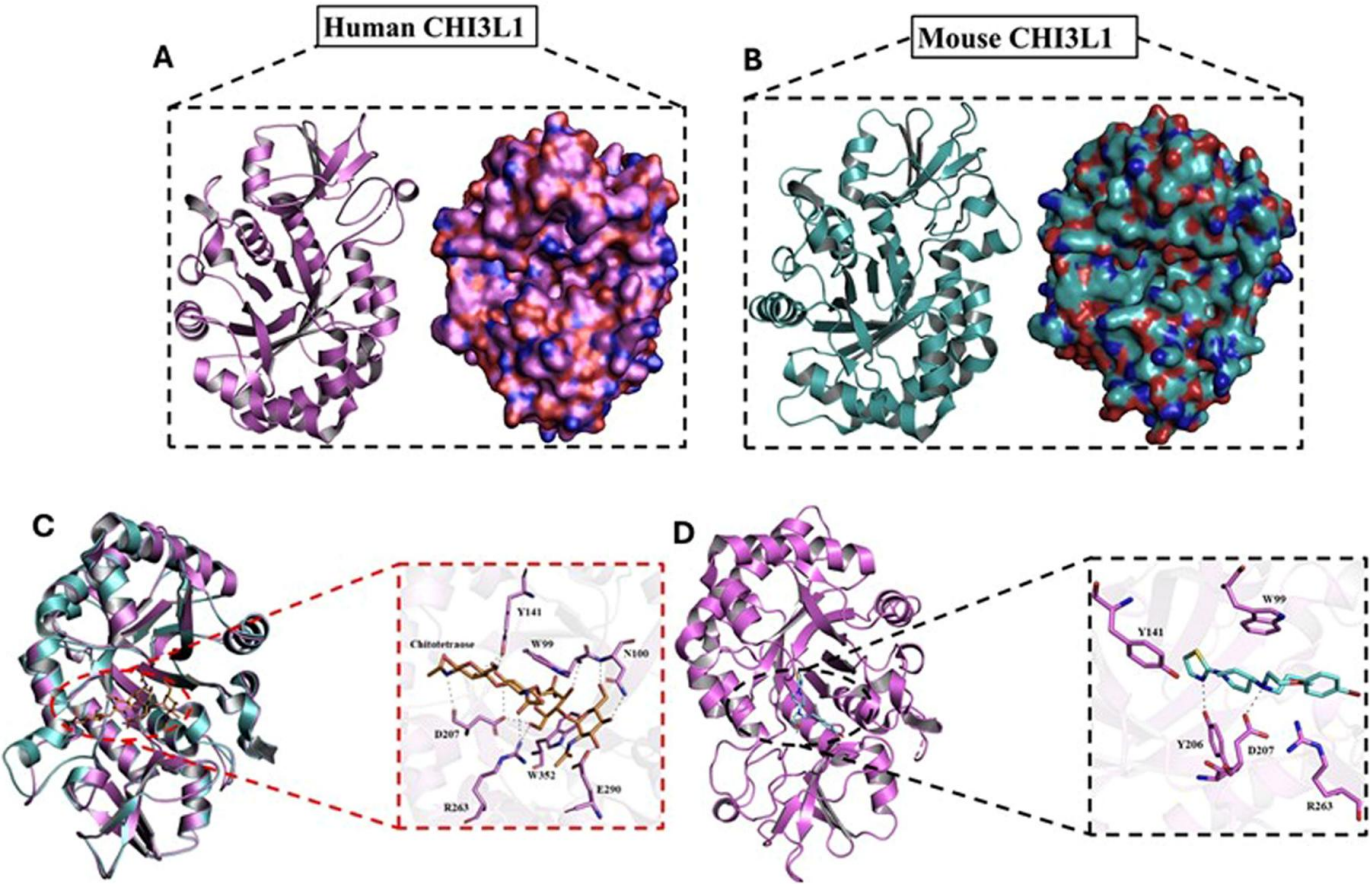
Crystal structures of human and mouse CHI3L1 and their conserved carbohydrate‐binding groove. (A, B) Structures of human and mouse CHI3L1. (C) Structural overlay showing bound chitotetraose within the carbohydrate‐binding cleft. (D) Modeled complex of CHI3L1 with inhibitor‐2 (PDB: 8R42), highlighting a potential drug‐binding pocket adjacent to the sugar‐binding groove. These structures reveal a highly conserved (β/α)_8_ barrel scaffold and ligand‐binding cleft, supporting CHI3L1's role as a structural sensor in innate immunity and inflammation. [Color figure can be viewed at wileyonlinelibrary.com]

Beyond architecture, post‐translational glycosylation further tunes CHI3L1's activity. Two N‐linked glycans, located at Asn60 and Asn273, stabilize folding and secretion while encoding receptor‐binding preferences [24]. Macrophage‐derived CHI3L1 displays complex‐type glycans, whereas epithelial cells secrete high‐mannose forms. This glycan code dictates whether CHI3L1 signals repair, fibrosis, or immune suppression [25]. Enzymatic removal of these glycans reduces receptor affinity, emphasizing their functional importance. Tumor‐associated complex‐type glycans favor immune suppression, while high‐mannose forms in fibrotic tissues drive fibroblast activation and matrix deposition. Because inflammation alters glycosylation machinery, this “glycan switch” dynamically links microenvironmental context to checkpoint activity.

Importantly, structural insights into CHI3L1 also provide a direct framework for therapeutic targeting. The extended carbohydrate‐binding groove and adjacent hydrophobic pockets form a chemically addressable surface that can accommodate small‐molecule ligands, as supported by recent structure‐guided modeling and inhibitor‐binding studies. In particular, the arrangement of residues such as Trp99, Trp352, and Asp207 defines a structurally conserved binding environment that supports both glycan recognition and small‐molecule engagement [26, 27]. The availability of high‐resolution crystal structures, including PDB entries such as 1HJW and more recent models (e.g., PDB: 8R42), enables structure‐guided drug design approaches to target this pseudoenzymatic scaffold [28]. These features position CHI3L1 as a tractable molecular target, where ligand binding to the pseudoactive groove or adjacent pockets can modulate CHI3L1‐mediated receptor interactions and downstream signaling pathways, thereby linking structural architecture directly to emerging therapeutic strategies targeting CHI3L1 signaling.

## 3 Regulation of CHI3L1 Expression

At the cellular level, CHI3L1 expression is finely regulated by cytokines, growth factors, extracellular‐matrix (ECM) signals, non‐coding RNAs, pharmacological agents, and environmental stressors [29]. Changes in ECM composition, particularly in chondrocytes, influence both synthesis and secretion of CHI3L1. In human chondrocytes and cartilage explants, IL‐1β and TGF‐β suppress CHI3L1 expression [30], whereas in epithelial cells (SW480, T84) IL‐1β and TNF‐α strongly induce its production [31]. In activated macrophages, IFN‐γ enhances CHI3L1 expression while IL‐4 inhibits it [32]. Pro‐inflammatory cytokines IL‐6, IL‐17, and IL‐18 further elevate secretion from chondrocytes [33], whereas IL‐7, IL‐11, IL‐12, PDGF, and bFGF exert minimal effects. Notably, IGF‐I and IGF‐II stimulate CHI3L1 synthesis in guinea‐pig but not human chondrocytes, indicating species‐specific regulation [34].

Beyond cytokine control, CHI3L1 is induced by a wide range of stress conditions—including hypoxia, ionizing radiation, DNA damage, oxidative stress, and nutrient deprivation—in U87 MG glioma cells, underscoring its role in cellular adaptation and survival [35]. Mechanistically, NF‐κB, MAPK/ERK, and PI3K/AKT pathways mediate transcriptional induction downstream of inflammatory and stress cues [20, 36, 37]. STAT3 activation further amplifies CHI3L1 transcription in cancer and fibrosis, forming feedback loops that reinforce immune suppression and tissue remodeling [38].

Recent studies identify the IL‐13Rα2–TMEM219–Galectin‐3 receptor triad as an upstream regulator of CHI3L1 expression. Engagement of this complex activates MAPK, Wnt/β‐catenin, and TGF‐β/Smad cascades, linking extracellular cytokine signals to transcriptional programs [20, 39, 40]. In parallel, epigenetic mechanisms—including histone acetylation and promoter methylation—fine‐tune CHI3L1 transcription in macrophages and glioma cells [40].

Finally, post‐translational and biophysical mechanisms add an additional layer of regulation. Surface‐plasmon‐resonance and ITC analyses reveal micromolar binding of CHI3L1 to chito‐oligosaccharides via a hydrophobic clamp formed by Trp31, Tyr34, and Trp352; alanine substitutions weaken binding, identifying these residues as structural anchors within the pseudoactive groove [41, 42]. These interactions indicate that CHI3L1 retains substrate‐recognition properties but redeploys them for structural tethering rather than catalysis. Predicted phosphorylation sites within the flexible N‐terminal region may regulate stability or secretion, while proteolytic cleavage in inflamed tissues generates truncated forms of unknown activity, suggesting that CHI3L1 operates as a conditionally gated checkpoint [30, 43]. Collectively, these insights define CHI3L1 as a checkpoint scaffold whose architecture and post‐translational modifications govern its stability, signaling, and druggability. The carbohydrate‐binding groove, glycan‐decorated epitopes, and heparin‐binding surface together provide multiple therapeutic interfaces for small‐molecule, antibody, or decoy‐based intervention [44]. High‐resolution cryo‐EM or integrative structural studies of CHI3L1 in complex with IL‐13Rα2, TMEM219, and Galectin‐3 will be crucial to connect molecular regulation with checkpoint function.

In summary, CHI3L1 expression is orchestrated through an integrated hierarchy of transcriptional, epigenetic, and post‐translational controls that respond to cytokine and stress cues. This multilayered regulation ensures that CHI3L1 is up‐regulated precisely under inflammatory, fibrotic, or hypoxic stress conditions where immune restraint and tissue repair intersect. The same adaptive logic that governs its expression underlies its next level of function—as a secreted checkpoint molecule that remodels immune and stromal signaling networks.

## 4 CHI3L1 as a Non‐Canonical Immune Checkpoint

The multi‐layered regulation of CHI3L1 expression—through cytokine, stress, and post‐translational controls—ensures that its production peaks precisely under conditions of inflammation, fibrosis, and metabolic stress. Once secreted, CHI3L1 does not merely mark these environments but actively shapes them, translating environmental signals into immunosuppressive outcomes. This transition from a stress‐responsive molecule to an extracellular immune modulator defines CHI3L1's identity as a non‐canonical checkpoint. CHI3L1 has been reported to function as a secreted, structurally distinct, pseudoenzymatic immune checkpoint that extends beyond the canonical PD‐(L)1 and CTLA‐4 pathways. Rather than acting through direct ligand–receptor engagement at the immune synapse, it has been reported to suppress T‐cell co‐stimulation, to up‐regulate inhibitory receptors, to enforce immune exclusion, and cooperates with classical checkpoints. From the extracellular milieu, CHI3L1 coordinates stromal, myeloid, and epithelial responses to hypoxia, tissue injury, and metabolic stress. It is mainly produced by fibroblasts, epithelial cells, macrophages, and other immune populations, and its activity depends on metabolic rewiring such as macrophage fatty‐acid oxidation and fibroblast‐driven fibrotic signaling [7]. By embedding checkpoint logic within tissue architecture, CHI3L1 extends immune regulation into the tissue‐metabolic domain, expanding the classical concept of checkpoint biology [11].

Within the tumor microenvironment, CHI3L1 has been reported to inhibit T‐cell co‐stimulatory molecules (ICOS, ICOSL, and CD28) while enhancing inhibitory checkpoints (B7‐1, B7‐2, CTLA‐4), thereby promoting metastasis [16]. Activation of the RIG‐I–like helicase (RLH) pathway reverses these effects, restoring co‐stimulation and limiting tumor spread [45]. In melanoma, CHI3L1 acts as a potent driver of progression and lymphatic dissemination by orchestrating an immunosuppressive transcriptional program that has been reported to induce PD‐L1, PD‐L2, PD‐1, LAG‐3, and TIM‐3, creating a multilayered barrier to antitumor immunity (Figure 3). CHI3L1 also has been reported to amplify IFN‐γ‐mediated PD‐L1 expression on macrophages, further reinforcing immune evasion; these effects can again be countered by RLH‐pathway activation [17]. Dual blockade of CHI3L1 and PD‐1/PD‐L1—particularly using bispecific antibodies—synergistically restores T‐cell infiltration by dismantling inhibitory signaling and stromal‐metabolic barriers, underscoring CHI3L1's central checkpoint role. In triple‐negative breast cancer, CHI3L1 has been reported to promote neutrophil recruitment and neutrophil‐extracellular‐trap (NET) formation, restricting CD8^+^ T‐cell entry; conversely, genetic deletion of Chi3l1 delays tumor onset and enhances immune‐checkpoint‐blockade (ICB) responsiveness [18].

**Figure 3:**
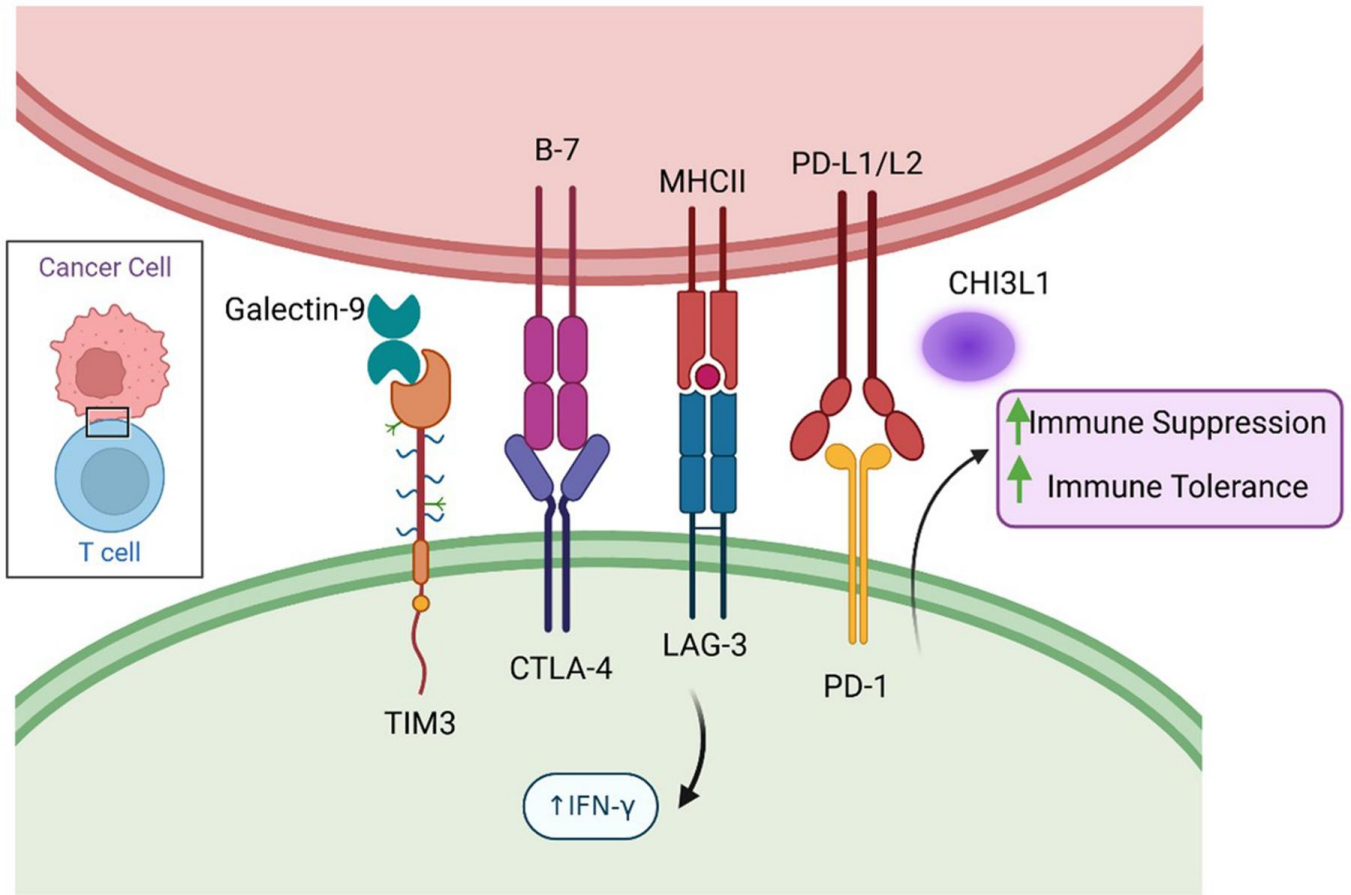
CHI3L1 enhances inhibitory checkpoint signaling to promote immune suppression and tolerance. In the tumor microenvironment, CHI3L1 upregulates inhibitory immune checkpoints, including B7–CTLA‐4, MHCII–LAG3, and PD‐L1/PD‐1, while amplifying IFN‐γ–driven PD‐L1 expression on cancer and myeloid cells. It also reinforces Galectin‐9–TIM3 signaling, collectively driving T‐cell exhaustion, immune suppression, and tolerance. Through these interactions, CHI3L1 acts as a central immune checkpoint–like molecule that strengthens tumor immune evasion and metastatic potential. [Color figure can be viewed at wileyonlinelibrary.com]

Beyond oncology, CHI3L1 has been reported to regulate immune balance in chronic inflammation and tissue remodeling. In COPD and pulmonary metastasis, it maintains epithelial survival and suppresses macrophage phagocytosis by activating “don't‐eat‐me” signals (CD47–SIRPα, CD24–Siglec10) and inhibiting “eat‐me” cues (calreticulin, HMGB1) via SHP‐1/2 [11] (Figure 4A). It further has been reported to recruit macrophages and neutrophils, polarizes them toward immunosuppressive phenotypes, and has been reported to induce IL‐6, IL‐8, and TGF‐β [46]. CHI3L1 has been reported to induce PD‐L1 expression, and is also embedded within STAT3‐associated signaling networks, contributing to self‐reinforcing suppressive circuits that link inflammation, fibrosis, and immune evasion [16, 38, 47].

**Figure 4:**
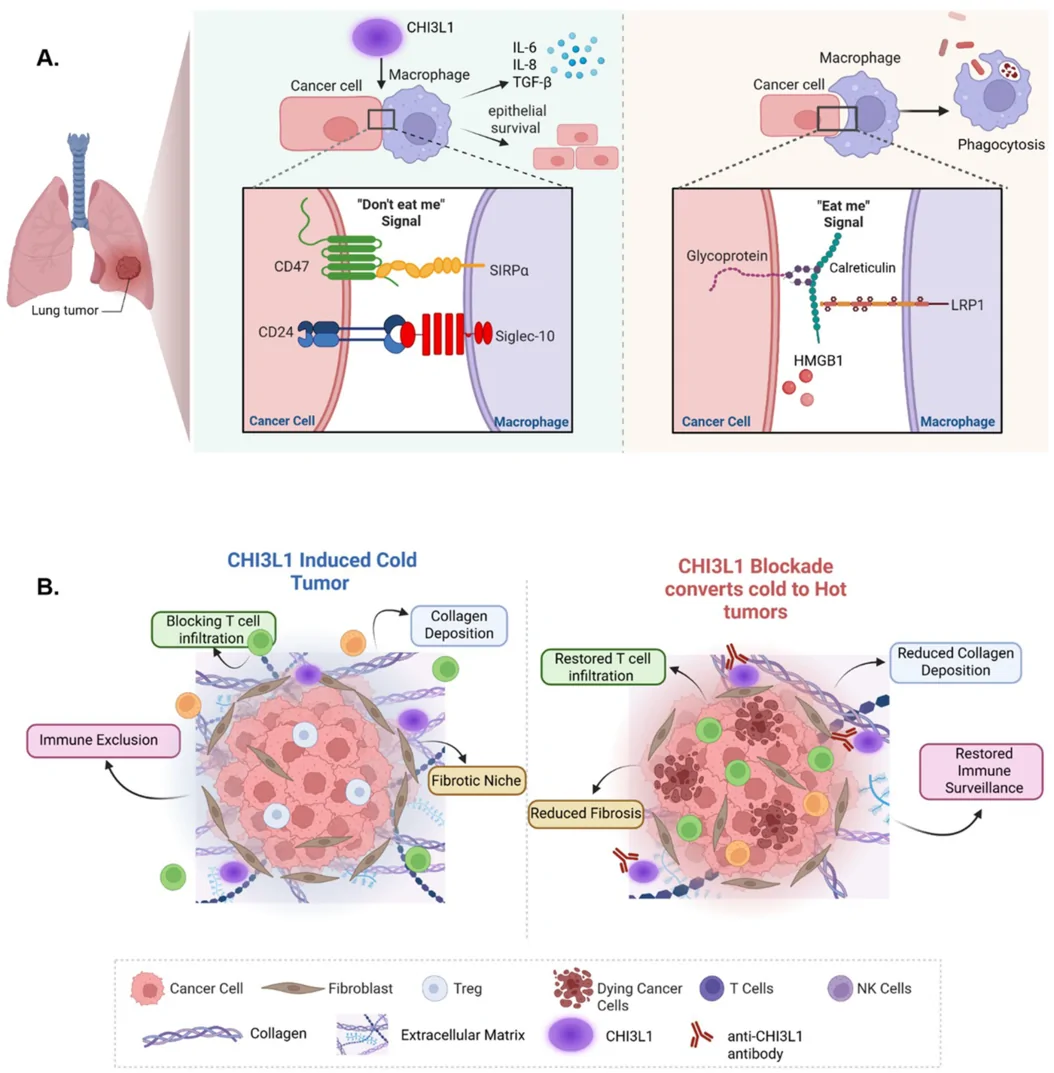
(A) CHI3L1 promotes cancer cell survival by inducing “don't eat me” signals and suppressing macrophage phagocytosis. In lung tumors, CHI3L1 secreted by cancer cells and macrophages enhances tumor cell survival by upregulating anti‐phagocytic “don't eat me” signals, including CD47–SIRPα and CD24–Siglec‐10 interactions, which inhibit macrophage‐mediated clearance. Concurrently, CHI3L1 stimulates macrophages to secrete IL‐6, IL‐8, and TGF‐β, further supporting epithelial survival and immune evasion. In contrast, loss or inhibition of CHI3L1 restores pro‐phagocytic “eat me” signals, such as calreticulin–LRP1 and HMGB1 release, leading to enhanced macrophage phagocytosis and tumor suppression. (B) CHI3L1‐driven immune exclusion and its reversal by CHI3L1 blockade. CHI3L1 has been reported to promote the formation of an immune‐excluded “cold” tumor microenvironment through fibroblast activation and excessive collagen deposition, creating a dense fibrotic niche that physically and biochemically restricts T cell infiltration. Elevated CHI3L1 levels reinforce immune evasion by sustaining extracellular matrix remodeling and suppressing immune cell trafficking into the tumor core. In contrast, therapeutic blockade of CHI3L1 with neutralizing antibodies reduces fibrosis and collagen deposition, thereby restoring T cell infiltration, reactivating immune surveillance, and converting immunologically “cold” tumors into “hot” ones responsive to immune attack. [Color figure can be viewed at wileyonlinelibrary.com]

The fibrotic arm of CHI3L1 signaling reinforces this suppressive network. By activating fibroblasts and driving collagen deposition, CHI3L1 stiffens the ECM [3]. The resulting rigidity not only restricts T‐cell infiltration but also activates YAP/TAZ mechanotransductive signaling, locking tissues into a state of chronic immune tolerance. CHI3L1‐enriched fibrotic niches—seen in melanoma, lung, and pancreatic cancers—are characterized by “cold,” non‐inflamed microenvironments with poor ICB responsiveness [48] (Figure 4B). Across these models, high CHI3L1 expression correlates with immune exclusion and metastasis, whereas its inhibition restores T‐cell infiltration, reduces fibrosis, and re‐establishes immune surveillance [49]. Even during infection and sepsis, CHI3L1 dampens T‐cell activation while sustaining macrophage viability, confirming its broader role as an immune suppressor integrating stromal and immune signals [50].

Together, these findings yield testable predictions: tumors with elevated CHI3L1 likely display ICI resistance due to sustained immune exclusion; targeting CHI3L1 could convert “cold” tumors into “hot,” ICI‐responsive ones; and in fibrotic or neuroinflammatory diseases where CHI3L1 is up‐regulated, its inhibition may restore immune surveillance and tissue homeostasis. While these studies provide a compelling mechanistic framework for CHI3L1‐mediated immune regulation, many of these findings originate from a focused body of work, and further independent validation will be important to establish the broader generalizability of these pathways. By extending checkpoint logic into extracellular, metabolic, and structural domains, CHI3L1 emerges as a tissue‐embedded, non‐canonical immune checkpoint that bridges immunity, fibrosis, and inflammation.

## 5 Receptor‐Interface Pharmacology and Structural Druggability

Understanding CHI3L1 as a non‐canonical immune checkpoint requires looking beyond the protein itself to the receptor machinery through which it signals. Unlike PD‐1 or CTLA‐4, which operate through fixed receptor–ligand pairs, CHI3L1 engages a modular, multi‐component receptor complex centered on IL‐13Rα2—once dismissed as a decoy receptor but now recognized as the hub of a structurally gated switch [51]. TMEM219, a transmembrane adaptor protein, forms a critical partnership with IL‐13Rα2 to transmit CHI3L1‐dependent signals. Upon CHI3L1 binding, IL‐13Rα2 has been reported to recruit TMEM219, whose cytoplasmic tail activates MAPK/Erk and PI3K/Akt pathways, inhibits apoptosis, has been shown to promote TGF‐β1 production, reinforces M2 macrophage polarization, drives fibroblast‐mediated collagen deposition, and enhances melanoma metastasis (Figure 5) [52]. Unlike canonical checkpoints with intrinsic signaling motifs, CHI3L1 delegates signal transduction to this receptor pair, making its suppressive activity dependent on the IL‐13Rα2–TMEM219 axis [53].

**Figure 5:**
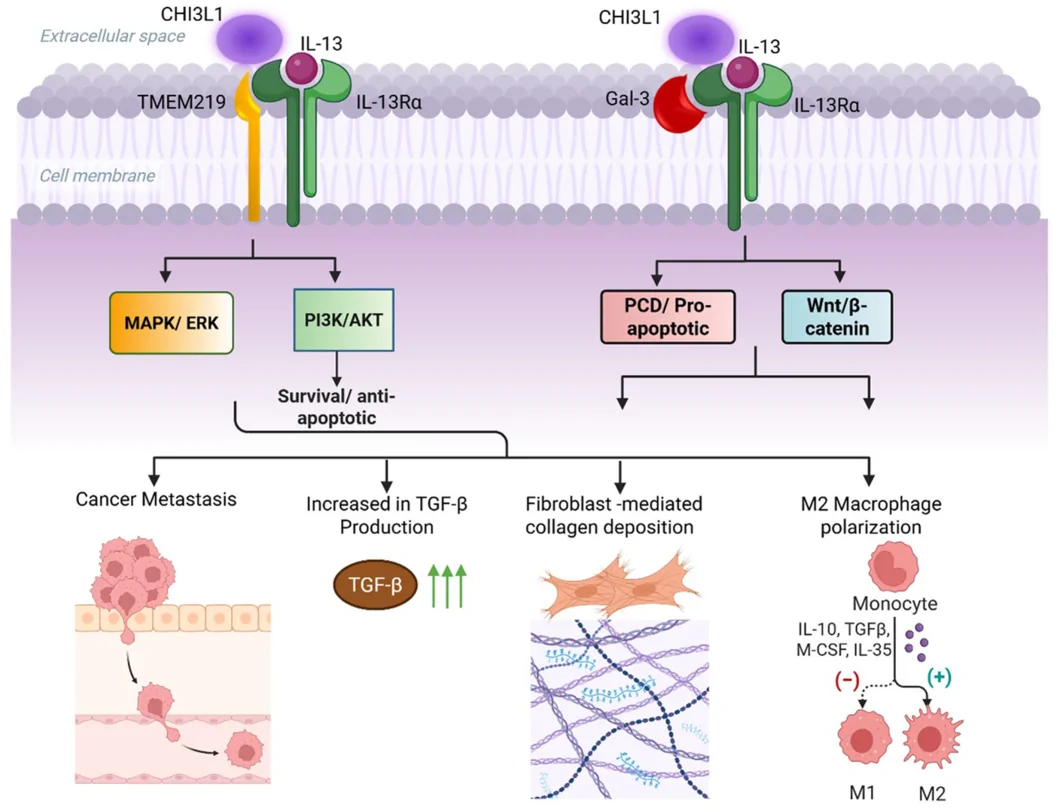
CHI3L1 receptor triad interactions and downstream signaling in fibrosis, immune modulation, and tumor progression. CHI3L1 engages distinct receptor complexes to regulate context‐dependent cellular outcomes. On the left, CHI3L1 binds the IL‐13Rα–TMEM219 complex, activating MAPK/ERK and PI3K/AKT pathways that promote cell survival, inhibit apoptosis, enhance TGF‐β production, and drive fibroblast activation and cancer metastasis. On the right, CHI3L1 interacts with the IL‐13Rα–Galectin‐3 (Gal‐3) complex, triggering pro‐apoptotic and differentiation‐related signaling via programmed cell death (PCD) and Wnt/β‐catenin pathways. These divergent signaling axes collectively mediate fibroblast‐driven collagen deposition, M2 macrophage polarization, and tumor immune evasion, establishing CHI3L1 as a key regulator at the intersection of immunity, fibrosis, and oncogenesis. [Color figure can be viewed at wileyonlinelibrary.com]

This signaling module, however, operates as a competitive interface rather than a binary switch. Galectin‐3 (Gal‐3)—a glycan‐binding lectin secreted by macrophages and fibroblasts—has been reported to compete with TMEM219 for IL‐13Rα2 binding [20]. This competition reshapes signaling outcomes: Gal‐3 reduces CHI3L1‐driven antiapoptotic effects in epithelial cells but enhances Wnt/β‐catenin activity in macrophages, amplifying fibroproliferative responses (Figure 5) [20]. In fibrotic tissues, Gal‐3 abundance tips the balance toward matrix deposition, whereas in tumors co‐expressing CHI3L1 and TMEM219, checkpoint signaling predominates. Thus, the IL‐13Rα2 interface has been reported to function as a biochemical toggle, TMEM219 directing suppression and Gal‐3 steering toward fibrosis, a balance that defines the pharmacology of CHI3L1 and its therapeutic manipulability.

Additional contextual amplifiers further modulate this axis. Syndecan‐1, heparan sulfates, integrins, and even CHI3L1's own glycans act as regulatory scaffolds that stabilize or bias signaling outcomes [54]. Syndecan‐1 anchors CHI3L1 in fibrotic stroma, prolonging receptor engagement; cell‐surface heparan sulfates enhance binding avidity through multivalent interactions; and integrins such as αvβ3 and αvβ5 connect CHI3L1 signaling to endothelial activation and angiogenesis [55]. Collectively, these interactions embed CHI3L1 within a wider extracellular framework that connects immune suppression to vascular and fibrotic remodeling.

A further layer of regulation arises from CHI3L1's glycan code. Two conserved N‐linked sites (Asn60 and Asn263) extend from its TIM‐barrel scaffold and determine receptor affinity and signaling potency [56]. Macrophage‐derived CHI3L1, enriched in complex‐type glycans, binds IL‐13Rα2 with higher avidity and elicits stronger TMEM219‐mediated signaling [52]. In contrast, epithelial‐derived high‐mannose glycoforms show altered secretion and binding, producing context‐dependent signaling diversity. These glycoforms effectively tune CHI3L1's output—biasing toward immune suppression, fibrosis, or angiogenesis depending on tissue context and receptor stoichiometry [24, 57].

This layered architecture provides multiple druggable seams. The CHI3L1–IL‐13Rα2 interaction can be disrupted by neutralizing antibodies or soluble decoys; the IL‐13Rα2–TMEM219 relay can be blocked by peptides or small molecules to silence checkpoint signaling without affecting IL‐13 biology; and Gal‐3–IL‐13Rα2 competition offers a therapeutic opening, as Gal‐3 antagonists in clinical trials for fibrosis and cancer may synergize with CHI3L1 inhibition [58]. Even CHI3L1's glycan structures themselves are potential targets—disease‐specific glycoforms could be selectively neutralized using lectin blockers or glycan‐mimetic antibodies.

## 6 CHI3L1 in Immunometabolism and Tissue Remodeling

CHI3L1's checkpoint function extends beyond receptor competition—it rewires the fundamental circuits of tissue homeostasis [59]. Rather than acting directly on T cells like canonical checkpoints, CHI3L1 exerts its influence indirectly by reprogramming macrophages, fibroblasts, and endothelial cells—the three gatekeepers that regulate immune access to tissue. Together, these cells form a coordinated “suppressive triad”: macrophages dictate the metabolic and antigen‐presenting landscape, fibroblasts build the structural and mechanical framework, and endothelial cells control vascular access and hypoxia. When CHI3L1 reshapes each component toward a suppressive state, the tissue itself becomes resistant to immune attack, regardless of antigenicity or costimulation.

### 6.1 Macrophages: Metabolic Reprogrammers of Immunity

CHI3L1 has been reported to skew macrophages away from inflammatory M1 programs toward M2‐like phenotypes characterized by arginase‐1, CD206, Ym1, FIZZ1, and MHC II expression, molecular hallmarks of macrophages specialized for tissue repair and immune regulation rather than pathogen clearance [5]. This shift is coupled to a metabolic reprogramming from glycolysis to fatty acid oxidation, producing ATP efficiently but depriving cells of the biosynthetic intermediates required for cytokine synthesis and reactive oxygen species production, functionally silencing inflammatory responses. The result is a macrophage population that suppresses CTL and NK cell activity, has been shown to promote T‐cell exhaustion, and secrete pro‐angiogenic and fibrotic mediators such as VEGF and TGF‐β to drive angiogenesis and metastasis. In breast and gastric cancers, CHI3L1‐expressing tumor‐associated macrophages (TAMs) enhance tumor survival through α4 integrin–VCAM‐1 interactions and PI3K/Akt signaling, creating a feedback loop that sustains immune privilege [60]. Similarly, in lung cancer, CHI3L1 has been reported to suppress key costimulatory signals (ICOS–ICOSL, CD28) while heightening inhibitory checkpoint pathways (CTLA‐4, PD‐L1), effectively constructing a metabolic and immunological firewall that shields tumors from immune attack [30].

### 6.2 Fibroblasts: Structural Amplifiers of Suppression

CHI3L1 has been reported to act as a potent activator of fibroblasts, which then undergo robust proliferation, accompanied by excessive deposition of collagen and other ECM components. The resulting ECM expansion not only alters tissue architecture but also increases matrix stiffness—a key physical cue that triggers YAP/TAZ‐dependent mechanotransduction loops [61]. These loops establish a self‐reinforcing circuit of fibrotic signaling, wherein mechanical tension perpetuates fibroblast activation even after the initial stimulus subsides. While mechanical stress itself reinforces immune exhaustion [62]. Beyond mechanical remodeling, CHI3L1 engages fibroblasts at the molecular level by binding to IL‐17RA, thereby activating the NF‐κB and MAPK cascades that drive myofibroblast differentiation. These contractile myofibroblasts secrete additional matrix proteins and pro‐fibrotic cytokines such as TGF‐β, further stiffening the microenvironment and deepening immune suppression [63]. In tumors, this manifests as immune‐excluded niches with dense, collagen‐rich barriers that physically prevent cytotoxic T cells from entering the tumor core. In fibrotic tissues, the same pathway sustains chronic scarring and tissue rigidity. Collectively, fibroblasts function as amplifiers of CHI3L1's checkpoint‐like effects, converting its biochemical signals into tangible, mechanical shields that couple persistent fibrosis with immune tolerance, ultimately sealing tissues in a state of pathological repair [64].

### 6.3 Endothelium: Vascular Gatekeepers and Hypoxic Enforcers

In endothelial cells, CHI3L1 has been reported to play a central role in vascular remodeling, contributing to the development of pro‐tumorigenic and fibrotic vascular networks. CHI3L1 has been reported to interact with syndecan‐1 and to modulate integrin‐associated signaling (αvβ3/αvβ5), contributing to activation of PTK2–MAPK–associated pathways and increased expression of angiogenic mediators such as VEGFR2. These effects are associated with altered vascular remodeling, including features of aberrant angiogenesis characterized by tortuous, leaky, and poorly perfused vasculature [65, 66]. Such vessels may support tumor growth while limiting immune cell infiltration, thereby contributing to nutrient‐rich but immune‐excluded microenvironments [19]. Beyond angiogenesis, CHI3L1 has also been reported to promote endothelial‐to‐mesenchymal transition (EndoMT), including through CD44–AKT/mTORC1‐associated signaling pathways. This phenotypic shift causes endothelial cells to lose their barrier function and acquire mesenchymal features, a transition that is a defining hallmark of vascular dysfunction in systemic sclerosis (SS) and other fibrotic disorders, linking CHI3L1 activity directly to tissue stiffening and chronic inflammation [67]. In melanoma, CHI3L1 reshapes the tumor secretome by upregulating VEGF‐A and CCL2, which attract immunosuppressive macrophages and exacerbate vessel leakiness [68]. The resulting hypoxic niche stabilizes HIF‐1α, which in turn amplifies CHI3L1 expression, creating a self‐perpetuating loop of angiogenesis, fibrosis, and immune evasion. In this way, CHI3L1 transforms vascular endothelium from a passive conduit into an active participant in immune suppression, structural remodeling, and disease persistence [69].

Altogether, CHI3L1 appears to establish a tri‐cellular checkpoint system: macrophages withdraw antigen presentation and rewire metabolism; fibroblasts fortify the ECM through YAP/TAZ loops; and endothelial cells remodel vasculature under HIF‐1α control. The cumulative outcome is a microenvironment where T cells face metabolic restriction, physical exclusion, and elevated activation thresholds. This explains why CHI3L1 expression correlates with PD‐1 dependency and resistance to PD‐1 blockade—removing a single inhibitory signal is futile if the broader suppressive architecture remains intact [17]. The same circuit manifests across contexts. In cancer, it produces immune exclusion, stromal fibrosis, and abnormal vasculature—key determinants of immunotherapy resistance. In fibrosis, macrophages, fibroblasts, and endothelium enact the same choreography absent malignancy, resulting in relentless scarring. During infection, CHI3L1 tempers immunopathology but delays pathogen clearance [70].

### 6.4 Context‐Dependent Functions of CHI3L1: Balancing Immune Suppression and Tissue Protection

A critical yet often under‐discussed aspect of CHI3L1 biology is the apparent contradiction between its immunosuppressive functions and its protective roles in acute inflammatory settings. While CHI3L1 has been implicated in promoting immune exclusion and checkpoint‐like signaling in cancer and fibrosis, several studies report that it attenuates immunopathology during infection by limiting excessive inflammation and preserving tissue integrity [3, 10]. This functional dichotomy highlights a core biological tension: mechanisms that dampen immune activation may be pathogenic in chronic disease but beneficial during acute inflammatory stress.

This duality is particularly evident in macrophage biology. CHI3L1‐associated skewing toward M2‐like phenotypes is frequently interpreted as immunosuppressive; however, M2 polarization is also central to resolution‐phase immunity, ECM remodeling, and tissue repair [25, 71]. Thus, CHI3L1‐driven macrophage reprogramming represents a temporally regulated process in which identical signaling outputs may have divergent consequences depending on disease stage and microenvironmental context.

Beyond immune modulation, CHI3L1 plays an important role in epithelial repair, angiogenesis, and maintenance of tissue integrity [72]. Experimental studies indicate that loss of CHI3L1 function has been associated with impaired wound healing and regenerative responses, suggesting that systemic inhibition may carry unintended consequences, particularly in tissues undergoing active repair or exposed to inflammatory stress [25, 73]. These findings raise important considerations for therapeutic targeting, where indiscriminate blockade may disrupt physiological homeostasis.

An additional layer of complexity arises from species‐specific differences in CHI3L1 biology. Many mechanistic insights derive from murine models, where ligand–receptor interactions and downstream signaling may not fully recapitulate human CHI3L1 function [12]. Such differences may influence both mechanistic interpretation and translational outcomes, underscoring the need for validation in human‐relevant systems.

Collectively, these observations position CHI3L1 not as a unidirectional pathological mediator but as a context‐dependent regulator at the interface of immune suppression and tissue protection. This duality has important implications for therapeutic design, suggesting that effective targeting strategies will need to incorporate spatial, temporal, and disease‐stage specificity to maximize efficacy while preserving physiological function—particularly in settings where immune suppression and tissue protection are tightly coupled.

## 7 Clinical and Preclinical Evidence for CHI3L1

The experimental and clinical literature on CHI3L1 has evolved from fragmented observations into a coherent body of evidence showing that it is not merely a biomarker but a mechanistic driver of immune suppression, tissue remodeling, and disease progression. Across fibrosis, cancer, neurodegeneration, infection, and wound healing, a consistent pattern emerges: CHI3L1 is induced in pathological states, actively sustains them, and its blockade disrupts these circuits. Importantly, interventional studies confirm that CHI3L1 is not just correlative but pharmacologically tractable, while biomarker analyses establish its reproducibility and prognostic value (Table 2).

### 7.1 CHI3L1 as a Checkpoint During Infectious Diseases

CHI3L1 has emerged as a pivotal host factor linking immune regulation, pathogen persistence, and tissue remodeling during infectious diseases. Elevated CHI3L1 expression is associated with poor outcomes in sepsis and severe respiratory infections such as tuberculosis and viral pneumonia, including COVID‐19, where it drives T cell dysfunction and has been shown to promote compensatory tissue repair that ultimately favors pathogen persistence [74, 75]. In COVID‐19, CHI3L1 has been reported to enhance ACE2 and Spike (S) priming protease expression, facilitating SARS‐CoV‐2 entry and infection across multiple variants of concern (VOCs) [76]. Inhibition of CHI3L1 by monoclonal antibodies or the small molecule kasugamycin significantly reduces pseudovirus infection, highlighting its potential as a broad‐spectrum antiviral target. In community‐acquired pneumonia (CAP), particularly respiratory syncytial virus (RSV) infection, CHI3L1 has been reported to promote M2 macrophage activation and Th2 polarization via PI3K/AKT and MAPK/ERK signaling [6, 77]. Neutralization of CHI3L1 attenuates Th2 cytokines and airway hyperresponsiveness while enhancing Treg responses [78, 79]. Similarly, in TLR‐mediated airway inflammation induced by viral and bacterial pathogens, CHI3L1 is upregulated via TNF‐α–dependent NF‐κB activation and is post‐transcriptionally regulated by miR‐149‐5p, underscoring tight control during epithelial immune responses [80].

CHI3L1 is also a key mediator of sepsis‐associated tissue injury and immune dysregulation. In an LPS‐induced hepatic sepsis model, CHI3L1 deficiency improved survival and reduced liver damage by selectively suppressing M2 macrophage polarization without affecting M1 responses, indicating that it has been shown to promote M2‐driven immunopathology [81]. Elevated CHI3L1 in septic skeletal muscle correlates with stem cell injury, while CHI3L1 silencing enhances cell proliferation, reduces apoptosis, and lowers proinflammatory cytokine levels [82]. These findings identify CHI3L1 as a regulator of sepsis‐induced organ injury and a promising host‐directed therapeutic target for limiting inflammatory damage. Collectively, these data position CHI3L1 as a central immunomodulator during infection—one that can be pharmacologically targeted to both temper excessive inflammation and restrict pathogen advantage.

### 7.2 Fibrosis and Fibroblast Remodeling

CHI3L1 is tightly linked to fibrotic remodeling across lung, liver, pancreas, and other tissues. The clearest proof of concept comes from preclinical inhibition studies. Su et al. developed fully human CHI3L1‐neutralizing antibodies (nAbs) that inhibited tumor growth, proliferation, and migration in lung, pancreatic, and colorectal cancer models [83]. In vivo, CHI3L1 nAbs reduced fibrosis and angiogenesis while restoring immune cell activity. Mechanistically, they suppressed pancreatic stellate cell activation and macrophage‐to‐myofibroblast transformation, highlighting their ability to attenuate fibroblast reprogramming and stromal hardening [12, 73]. In bleomycin‐induced pulmonary fibrosis, neutralizing monoclonal antibodies against CHI3L1 reduced fibroblast proliferation and collagen deposition by ~40%–50% and improved lung compliance to levels comparable with pirfenidone (forced vital capacity preservation ~38% vs. ~35% with pirfenidone) [84]. Inhaled siRNAs silenced CHI3L1 transcripts by > 70%, reversed airway remodeling, and restored tissue mechanics in murine lungs, while antisense oligonucleotides attenuated airway thickening in asthma models [85]. Soluble IL‐13Rα2 decoys, by intercepting CHI3L1 before TMEM219 recruitment, curtailed ECM accumulation and disrupted fibroblast activation loops [52].

Clinical biomarker data parallel these interventional findings. In idiopathic pulmonary fibrosis (IPF), serum CHI3L1 predicts forced vital capacity decline with hazard ratios of 1.8–2.2 across independent cohorts, outperforming markers such as KL‐6 and surfactant protein D [86]. PF patients with persistently high CHI3L1 despite pirfenidone or nintedanib experience accelerated decline (> 200 mL/year vs. < 100 mL/year in CHI3L1‐low patients), defining a biomarker‐enriched subgroup for upstream CHI3L1 blockade [87]. In cirrhosis and chronic kidney disease, CHI3L1 correlates with progressive loss of function, reinforcing its pan‐fibrotic relevance [88].

### 7.3 Cancer and Immune Exclusion

In oncology, CHI3L1 has been reported to function as a stromal checkpoint that enforces immune exclusion [18]. In breast and lung xenograft models, anti‐CHI3L1 antibodies suppressed angiogenesis and significantly reduced metastatic spread [89]. In colon cancer, CHI3L1 neutralization inhibited tumor‐cell proliferation and migration while promoting apoptosis in a dose‐dependent manner [90]. Mechanistically, CHI3L1 interacts with VEGFA and is linked to NAMPT‐dependent metabolic regulation; combined inhibition of CHI3L1, VEGFA, and NAMPT further reduced tumor cell viability, ATP and NADH levels, and enhanced apoptosis [90]. In a breast cancer model, anti‐CHI3L1 antibody treatment suppressed tumor growth by blocking CHI3L1‐mediated activation of the MAF/CTLA‐4 axis, restoring CD8^+^ T cell infiltration and countering immune evasion [91]. Separately, the small‐molecule CHI3L1 inhibitor K284 markedly suppressed lung metastasis and tumor growth in both melanoma and lung cancer models [91]. Mechanistically, K284 binds to the chitin‐binding domain of CHI3L1, blocks its interaction with IL‐13Rα2, and inhibits downstream JNK–AP‐1 signaling [92].

Additional work has identified CHI3L1 as a mediator of direct immune effector suppression. Abbass Darwich et al. showed that CHI3L1 impairs cytotoxic T cell and NK cell granule machinery, inhibiting perforin and granzyme release and thereby reducing tumor cell killing while preserving immune cell viability [93]. CHI3L1 blockade restored cytotoxic function in vitro and in vivo. In melanoma, CHI3L1 has been reported to suppress T cell co‐stimulation (ICOS/ICOSL, CD28/B7) while enhancing CTLA‐4 and its ligands and upregulating PD‐L1, thereby driving metastatic spread [16]. Activation of innate pathways or direct blockade of CHI3L1 restored co‐stimulation, reduced checkpoint signaling, and limited metastasis. Combination therapies targeting CHI3L1 alongside PD‐1 or CTLA‐4—particularly via bispecific antibodies—produced synergistic antitumor effects, enhanced T cell–mediated cytotoxicity, and upregulated tumor suppressors such as PTEN [17, 94]. Bispecific PD‐1×CHI3L1 antibodies restored CD8^+^ infiltration and converted partial responses into durable regressions in melanoma [84], conceptually paralleling the logic of dual PD‐1×LAG‐3 and PD‐1×CTLA‐4 regimens in the clinic (e.g., RELATIVITY‐047, NCT03470922; CheckMate‐067, NCT01844505), where multi‐checkpoint targeting improves response durability. Clinically, high CHI3L1 expression in glioblastoma, colorectal carcinoma, pancreatic adenocarcinoma, and NSCLC correlates with poor survival and resistance to PD‐1 blockade [89]. In NSCLC cohorts, elevated serum CHI3L1 predicted shorter overall survival (HR ~ 2.1, 95% CI 1.5–2.9) [95, 96]. Together, these results position CHI3L1 as both a mechanistic driver and a stratification marker in oncology.

### 7.4 Neuroinflammation, Acute, and Systemic Conditions

CHI3L1 is among the most reproducible cerebrospinal fluid biomarkers of glial activation [97]. Alzheimer's disease (AD), the leading cause of dementia, typically manifests later in life, with prevalence doubling every 5 years after age 65, Its pathology is driven by activated microglia and astrocytes, which influence β‐amyloid‐42 (Aβ42) production and clearance, exacerbating tau pathology and neurodegeneration [98, 99]. In AD, elevated CSF CHI3L1 correlates with amyloid burden and cognitive decline (HR ~ 1.7 for progression from mild cognitive impairment to AD. Elevated CHI3L1 levels in cerebrospinal fluid (CSF) and brain tissues correlate with cognitive decline and mild AD‐type dementia, highlighting its potential as a diagnostic biomarker [58, 100, 101]. Mechanistically, CHI3L1 modulates microglial activation and phagocytic efficiency, impairing Aβ clearance and thereby promoting plaque accumulation and aggregation; correspondingly, elevated CSF CHI3L1/Aβ42 ratios serve as predictive biomarkers of cognitive decline and AD progression [102, 103, 104].

Positron emission tomography (PET) studies show that elevated CSF CHI3L1 (YKL‐40) levels parallel tau burden and reflect regional astrocyte activation; although associations with Aβ burden are less consistent, the marker nevertheless indexes reactive astrogliosis heterogeneity and correlates with disease progression [105, 106]. Moreover, CHI3L1 levels associate with structural brain changes, including axonal and synaptic damage, reinforcing its role as both a biomarker and a potential therapeutic target in AD [58, 107]. Similarly, CHI3L1 secreted by astrocytes impairs hippocampal neurogenesis in AD by activating the CRTH2 receptor and downstream IKKβ–S6K1–S6 signaling, reducing neural stem cell proliferation and neuronal differentiation [58]. Using human iPSC‐derived neurogenesis models and astrocyte‐specific CHI3L1 knockout in 5XFAD mice, inhibition of CHI3L1 or blockade of CRTH2 signaling restored neurogenesis and improved cognitive function. Together, these findings identify CHI3L1 as a central effector of astrocyte‐driven neurodegeneration, linking neuroinflammatory signaling to amyloid burden and cognitive deterioration, and highlight it as a tractable target for therapeutic intervention in AD. In multiple sclerosis, longitudinal studies show that higher CSF CHI3L1 predicts conversion from clinically isolated syndrome to definite MS (HR ~ 2.4, *p* < 0.01) [108], outperforming neurofilament light chain in some cohorts. In traumatic brain injury, CHI3L1 rises acutely within 24 h and tracks with astrocytic activation, making it a sensitive early neuroinflammatory marker [109]. These reproducible CNS data suggest CHI3L1 could serve both as a diagnostic biomarker and as a pharmacodynamic readout in neuroimmunology trials. In the 5xFAD mouse model of AD, which expresses human amyloid precursor protein and presenilin 1 transgenes, elevated levels of chitinase 3‐like‐1 (CHI3L1) have been observed in the hippocampus by 9 months of age [110]. Mechanistically, CHI3L1 upregulation parallels the activation of the NF‐κB signaling pathway—likely driven by astrocytic and microglial inflammatory cascades that CHI3L1 itself amplifies through autocrine and paracrine feedback. NF‐κB activation subsequently enhances transcription of β‐site amyloid precursor protein‐cleaving enzyme 1 (BACE1), the rate‐limiting enzyme in amyloid‐β (Aβ) generation [111]. This CHI3L1–NF‐κB–BACE1 axis thus provides a mechanistic link between neuroinflammation and amyloidogenesis. Inhibition of NF‐κB signaling reduces *BACE1* expression, lowers Aβ burden, and ameliorates memory deficits and neurodegeneration in vivo, highlighting CHI3L1‐induced inflammatory signaling as a potential therapeutic entry point in AD.

In sepsis, serum CHI3L1 is consistently elevated in non‐survivors, predicting 28‐day mortality with hazard ratios of 1.9–2.3 independent of age and SOFA score [112, 113]. Mechanistically, elevated CHI3L1 reflects suppression of T‐cell activation and sustained macrophage survival, a maladaptive circuit that limits pathogen clearance. During COVID‐19 pneumonia, serum CHI3L1 levels rose sharply in severe disease, correlating with hypoxemia and inflammatory burden; patients in the highest quartile of CHI3L1 had a > 2‐fold risk of ICU admission or death [114]. These observations extend CHI3L1's checkpoint logic into acute inflammatory contexts, showing that it functions across both chronic and acute disease states.

Localized data in rheumatoid arthritis (RA) and inflammatory bowel disease (IBD) provide further mechanistic anchors. In RA, synovial fluid CHI3L1 concentrations closely track joint inflammation, correlating with DAS28 activity scores (*r* = 0.65–0.7) [115]. In IBD, serum CHI3L1 is elevated during flare and associates with histological markers of remodeling and fibrosis [116]. These settings emphasize that CHI3L1 is not a generic inflammation marker but a tissue‐embedded regulator of fibrosis, angiogenesis, and immune suppression. A critical translational consideration is safety. CHI3L1‐null mice exhibit delayed cutaneous closure and impaired neovascularization, highlighting its physiological role in epithelial repair [10]. This liability mandates cautious trial design: systemic inhibition could impair normal healing, requiring mitigation through local delivery, intermittent dosing, or glycoform‐specific targeting. These preclinical findings therefore serve as safety anchors, ensuring that therapeutic innovation is paired with mechanistic foresight.

## 8 Medicinal Chemistry and Structure‐Guided Targeting of CHI3L1

The small‐molecule targeting of CHI3L1 has progressed from isolated tool compounds to multiple chemically distinct hit and lead series that collectively validate the glycan‐ or oligosaccharide‐binding cleft as a druggable surface on this nonenzymatic protein. Although CHI3L1 lacks catalytic activity, several orthogonal discovery approaches, including structure‐based design, pharmacophore‐guided screening, SPR‐based high‐throughput screening, and ultralarge virtual screening, have identified ligands that bind this cleft and translate target engagement into measurable downstream functional effects, most consistently inhibition of STAT3‐associated signaling in glioblastoma‐relevant models. To better organize these advances, current CHI3L1‐targeting strategies can be grouped into distinct chemical and mechanistic categories.

### 8.1 Chemical Classes Targeting CHI3L1

From a medicinal chemistry standpoint, the current CHI3L1 landscape can be divided into four practical groups. The first is the early structure‐based reference scaffold represented by K284 (Table 1), which established that small molecules can bind the chitin‐binding region of CHI3L1 and, in that specific series, block the CHI3L1–IL‐13Rα2 interaction [117]. The second is the K284‐derived optimization lineage, in which systematic scaffold modification produced compounds such as **11g** and later **10p** (Table 1) with markedly improved affinity, solubility, pharmacokinetic behavior, and activity in multicellular GBM spheroid systems [118]. The third group comprises pharmacophore‐derived hits such as compounds **8** and **39** (Table 1), which were identified from large‐scale virtual screening and validated by MST and SPR [119]. The fourth includes chemically distinct screening‐derived hits such as **1–4**, **1–7**, and **9e** (Table 1), identified by SPR‐HTS or ultralarge chemical space screening, which broaden the available chemotypes for CHI3L1 modulation [120].

**Table 1:** Representative CHI3L1 small‐molecule inhibitors and their pharmacological profiles.

Compound	Series/Class	Binding site/key interaction logic	Main functional evidence	Development status
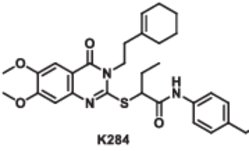	Early structure‐based reference scaffold	Binds CHI3L1 chitin/glycan‐binding region; reported to block CHI3L1–IL‐13Rα2 interaction	Anti‐metastatic and anti‐inflammatory activity reported in prior disease models; reference scaffold for optimization	Preclinical reference scaffold [117]
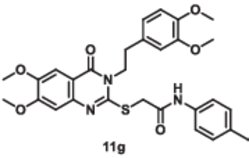	Optimized K284‐derived lead	Preserves core cleft binding; improved docking profile vs K284 without unfavorable contacts	Improved solubility, permeability, microsomal/plasma stability, lower PPB, improved hERG, stronger GBM spheroid activity and reduced pSTAT3	Optimized lead [118]
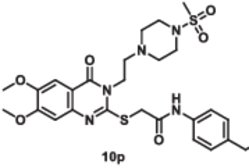	Advanced optimized analogue from 11 g lineage	Stable cleft engagement supported by docking/MD; interactions include Asp207, Trp99, Tyr141, Trp352, Met204, Phe261, Glu290	Major solubility gain, improved stability, no detectable hERG up to 100 μM, stronger 3D GBM spheroid efficacy	Advanced lead candidate [118]
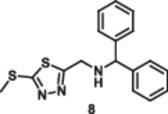	Pharmacophore‐derived hit	Glycan‐binding cleft; Trp99 conformational stabilization plus Tyr206 interaction	Reduced GBM spheroid viability, decreased pSTAT3, antiangiogenic activity	Validated hit/early lead [119]
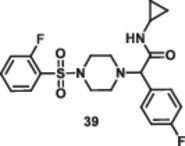	Pharmacophore‐derived hit	Glycan‐binding cleft; same general binding logic as 8 but weaker	More modest inhibition of spheroid viability and pSTAT3 than compound 8	Validated hit [119]
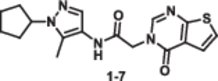	SPR‐HTS hit	CHI3L1 cleft binder; also inhibits CHI3L1–galectin‐3 interaction	Inhibits CHI3L1–galectin‐3, reduces GBM spheroid viability, suppresses pSTAT3; favorable selectivity profile in reported assays	Validated hit/early lead [120]
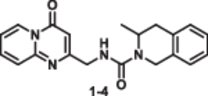	SPR‐HTS hit	CHI3L1 cleft binder; inhibits CHI3L1–galectin‐3 interaction	Active in GBM spheroids and Gal‐3 assay, but weaker overall than 1–7	Validated hit [120]
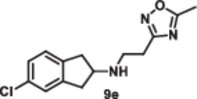	Ultralarge‐screening lead‐like hit	Cleft binder engaging central polar residues with remaining unoccupied optimization space near E290/M204/M350	Modest Gal‐3 interference, reduced GBM spheroid viability, reduced pSTAT3, phenocopied CHI3L1‐neutralizing antibody behavior	Lead‐like hit/hit‐to‐lead starting point [12074

**Table 2:** Mechanistic and translational evidence for CHI3L1 across disease contexts.

Disease context	Experimental/clinical model	Intervention/Observation	Mechanistic readout	Translational anchor
Pulmonary fibrosis	Bleomycin‐challenged mouse	Anti‐CHI3L1 mAb	↓ Fibroblast proliferation, ↓ collagen deposition, ↓ ECM	Comparable efficacy to pirfenidone (FVC preservation)
Pulmonary fibrosis	Mouse (inhaled delivery)	siRNA against CHI3L1	↓ CHI3L1 transcripts, ↓ fibroblast activation, reversal of airway remodeling	Feasible local therapy with minimized systemic risk
Asthma	Murine asthma model	Antisense oligonucleotide	Attenuated airway remodeling, ↓ fibroblast activation	RNA‐based targeting validated in vivo
Pulmonary fibrosis	Mouse	Soluble IL‐13Rα2 decoy	Blocked TMEM219 relay, ↓ ERK signaling, ↓ ECM deposition	Receptor interface shown druggable
Cancer (breast, lung)	Xenograft	Anti‐CHI3L1 mAb	↓ Angiogenesis, ↓ metastasis	Validates CHI3L1 as metastatic niche regulator
Cancer (colon, lung)	Knockout mouse	CHI3L1 deficiency	Restored CD8^+^ T‐cell infiltration, ↓ tumor growth	Genetic evidence of checkpoint logic
Melanoma metastasis	Mouse	Small molecule K284	Bound pseudoactive groove, ↓ dissemination to lung/liver	Proof‐of‐principle small‐molecule druggability
Cancer (melanoma)	Mouse	Bispecific PD‐1 × CHI3L1 antibody	Restored CD8^+^ infiltration, durable regression	Mirrors PD‐1 × LAG‐3 bispecific trial precedents
Glioblastoma	Patient tumor (IHC/ELISA)	High CHI3L1 expression	Correlated with poor OS, PD‐1 resistance	Prognostic marker + stratification tool
NSCLC	Patient serum	ELISA	High circulating CHI3L1 → shorter OS (HR ~ 2.1)	Companion diagnostic potential
Colorectal cancer	Patient tissue	IHC/ELISA	High CHI3L1 expression linked to immune exclusion	Clinical evidence for stromal checkpoint role
IPF	Human cohort	Serum CHI3L1	Predicted FVC decline (HR 1.8–2.2)	Clinical enrichment biomarker
Cirrhosis	Patient serum	ELISA	Elevated CHI3L1 linked with worsening function	Pan‐fibrotic marker
CKD	Patient plasma	ELISA	Elevated CHI3L1 predicts renal progression	Extends checkpoint logic beyond oncology
Alzheimer's disease	Human CSF	ELISA	↑ CHI3L1 correlates with amyloid burden, cognitive decline	Reproducible CNS biomarker
Multiple sclerosis	Human CSF	Longitudinal cohort	Elevated CHI3L1 predicts conversion to MS (HR ~ 2.4)	Stratification and disease progression marker
Traumatic brain injury	Human CSF	Acute measurement	Rapid rise in CHI3L1, correlated with astrocytic activation	Sensitive early neuroinflammation marker
Sepsis	Patient plasma	Observational	High CHI3L1 → ↑ 28‐day mortality (HR ~ 1.9–2.3)	Independent prognostic biomarker
COVID‐19 pneumonia	Patient serum	Observational	↑ CHI3L1 in severe cases, correlated with hypoxemia	Stratification tool in acute infection
Wound healing	CHI3L1‐null mouse	Knockout	Delayed closure, impaired angiogenesis	Safety anchor for clinical trials

### 8.2 Binding Mode and Structural Determinants

Despite this chemical diversity, the structure–activity relationships converge on a common interaction logic within the glycan‐binding cleft. A recurring feature in active ligands is the presence of a positively ionizable center, often embedded in morpholine‐, piperazine‐, or tertiary amine‐containing motifs, positioned to engage Asp207 and to stabilize Trp99 through π‐cation interactions. In the pharmacophore‐derived series, docking explicitly showed that compounds **8** and **39** stabilize Trp99 in a conformation unfavorable for oligosaccharide binding, indicating that Trp99‐centered conformational control is a major determinant of productive binding. A second recurring interaction is directional hydrogen bonding to residues such as Tyr206, or in some series Tyr141 or related polar residues, which helps orient the ligand in the cleft. Together, these polar anchoring interactions are reinforced by occupation of a multipart hydrophobic landscape within the pocket, including a central lipophilic subpocket and adjacent conserved hydrophobic regions.

### 8.3 Structure–Activity Relationships (SAR)

The pharmacophore‐derived series illustrates this binding logic clearly, with compound 8 displaying higher affinity for CHI3L1 than compound **39**, consistent with improved engagement of the predicted binding pocket and supporting the proposed structure–activity relationship [119]. Docking showed that both compounds combine a charged nitrogen that supports Trp99‐centered binding with additional interactions in the central lipophilic subpocket; compound **8** also showed stronger functional consequences, including clearer suppression of phospho‐STAT3 and more pronounced reduction of GBM spheroid viability. These results support the view that the balance of polar anchoring and hydrophobic subpocket occupancy, rather than simple lipophilicity alone, governs productive CHI3L1 binding in this series.

A similarly informative SAR emerges from the K284 optimization lineage. In the first optimization step, compounds **11a** and **11b** (Figure 6), which introduced morpholine‐containing replacements, failed to show measurable CHI3L1 binding, indicating that adding polarity without preserving the correct aromatic and spatial features was insufficient [44]. Compounds **11c**–**11e** (Figure 6), which introduced bulky hydrophobic replacements, also failed to improve activity. In contrast, compound **11f** showed measurable binding, and addition of the 3,4‐dimethoxyphenyl moiety in **11g** improved affinity substantially (Figure 6) [44]. A second SAR layer involved the distal 4‐ethylphenyl fragment: the unsubstituted analogue **11h** was weaker, para‐methoxy analogue **11i** weakened further, para‐chloro **11j** lost measurable binding, and ortho/meta substitution patterns also abolished dose‐responsive interaction (Figure 6). These data support two conclusions: first, the 3,4‐dimethoxyphenyl‐containing region is a productive replacement in the CHI3L1 cleft, and second, the para‐ethyl arrangement on the distal phenyl ring is important for maintaining high‐affinity binding in this scaffold family.

**Figure 6:**
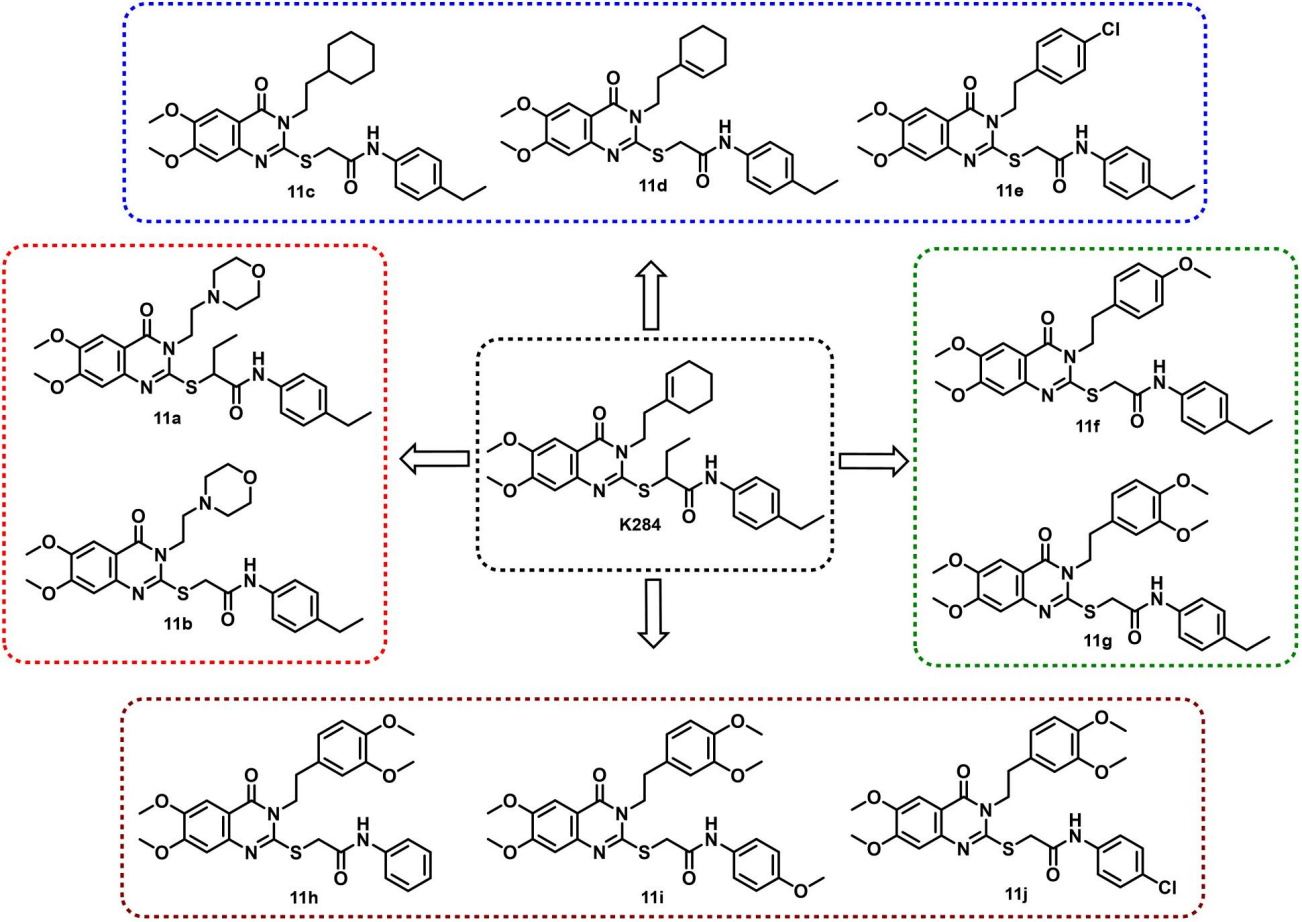
Series of chemical optimization of K284 scaffold as a CHI3L1 binder to give insight into the SAR of CHI3L1 binding. [Color figure can be viewed at wileyonlinelibrary.com]

### 8.4 Lead Optimization and Developability

That same K284‐derived series also provides the strongest direct connection between medicinal chemistry and developability. Relative to K284, compound **11g** improved kinetic solubility, PAMPA permeability, plasma and microsomal stability, reduced plasma protein binding, and showed a more favorable hERG profile. Functionally, **11g** reduced viability, spheroid mass, migration, and pSTAT3 levels in multicellular 3D GBM spheroids more effectively than K284. These data move the series beyond binding optimization alone and show that CHI3L1‐targeted medicinal chemistry can yield compounds with simultaneous improvements in target engagement, in vitro ADME behavior, and disease‐relevant cellular activity.

The subsequent piperazine‐focused refinement that yielded 10p further strengthens this medicinal chemistry narrative. In that series, the 3,4‐dimethoxyphenethyl‐containing region identified in **11g** was treated as a modifiable zone for improving physicochemical behavior rather than simply maximizing potency. Compound **10p** retained comparable affinity to **11g**, but dramatically improved kinetic solubility, increased microsomal and plasma half‐lives, and showed no detectable hERG inhibition [121]. Docking and MD simulations in that study supported stable pocket engagement involving residues such as Asp207, Trp99, Tyr141, Trp352, Met204, Phe261, and Glu290, suggesting that further optimization of this lineage can improve developability without sacrificing the core CHI3L1 interaction pattern. Functionally, **10p** also outperformed earlier analogues in 3D GBM spheroid assays, supporting its designation as a more advanced lead candidate within this scaffold family. Notably, compounds such as **11g** and **10p** represent advanced preclinical candidates, combining improved pharmacokinetic properties with robust efficacy in disease‐relevant models, including 3D glioblastoma spheroids.

### 8.5 Emerging Chemotypes From Screening Campaigns

The SPR‐HTS and ultralarge‐screening studies contribute additional mechanistically useful chemotypes. In the SPR‐based campaign, compounds **1–4** and **1–7** were confirmed as binders, and both inhibited the CHI3L1–galectin‐3 interaction, with **1–7** again showing the stronger overall profile [120]. The same study linked binding kinetics to screening quality, showing that compounds with rapid association and clean dissociation behavior were more likely to represent true binders than compounds with persistent sensorgram residuals. In the ultralarge‐screening study, compound **9e** emerged as a lead‐like hit but modest interference with CHI3L1–galectin‐3 interaction, and dose‐dependent suppression of GBM spheroid viability and pSTAT3. Importantly, pocket analysis around **9e** highlighted unoccupied space near Glu290 and Met204/Met350, explicitly identifying vectors for further potency optimization. These two studies therefore expand the available chemical space around CHI3L1 and show that the target can support multiple independent hit series beyond the K284 lineage.

Taken together, these studies establish a balanced medicinal chemistry framework for CHI3L1 targeting. Productive ligands combine polar anchoring to residues such as Asp207, Trp99‐centered conformational control, directional hydrogen bonding to residues such as Tyr206 or Tyr141, and occupation of a multipart hydrophobic cleft. The field has now progressed from initial binders to optimized leads with improved ADME properties and robust activity in complex 3D cellular models. This makes CHI3L1 not merely a conceptual target, but an increasingly mature platform for structure‐guided small‐molecule discovery in cancer and neuroinflammatory disease contexts.

## 9 Therapeutic Targeting of CHI3L1: Multimodal Strategies and Clinical Translation

Beyond the structure–activity relationships governing small‐molecule binding described above, CHI3L1‐targeting strategies must be evaluated in a broader translational context, encompassing biologics, RNA‐based approaches, and in vivo pharmacological modulation. CHI3L1 is emerging as a non‐canonical checkpoint with therapeutic potential, supported by interventions using antibodies, small molecules, RNA, and receptor decoys [10, 122]. These approaches demonstrate that CHI3L1 is highly druggable, and its blockade can modulate disease outcomes in cancer, fibrosis, infection, and neuroinflammation. The key challenge now is optimizing its integration into therapies that maximize benefit while minimizing risk.

Monotherapies targeting CHI3L1 have provided the first experimental validation of its therapeutic tractability. Among these, neutralizing antibodies represent the most advanced modality. In pulmonary fibrosis models, CHI3L1 blockade with monoclonal antibodies suppresses fibroblast proliferation, collagen deposition, and aberrant ECM remodeling (Figure 7, left panel), achieving efficacy comparable to pirfenidone in bleomycin‐challenged mice [83]. In oncology, antibody‐mediated inhibition of CHI3L1 curtails angiogenesis and metastatic spread in breast and lung xenograft models, reinforcing its role in constructing the pro‐metastatic niche [52]. Mechanistically, these benefits extend beyond antibody binding itself, soluble IL‐13Rα2 decoys mimic the neutralizing effect by sequestering CHI3L1 before it has been reported to recruit TMEM219, thereby preventing ERK pathway activation and restoring apoptotic sensitivity in epithelial cells [89, 123]. Collectively, these results establish that direct interference with CHI3L1 or interception through its receptor decoys can effectively dismantle pathogenic signaling across fibrotic and neoplastic settings.

**Figure 7:**
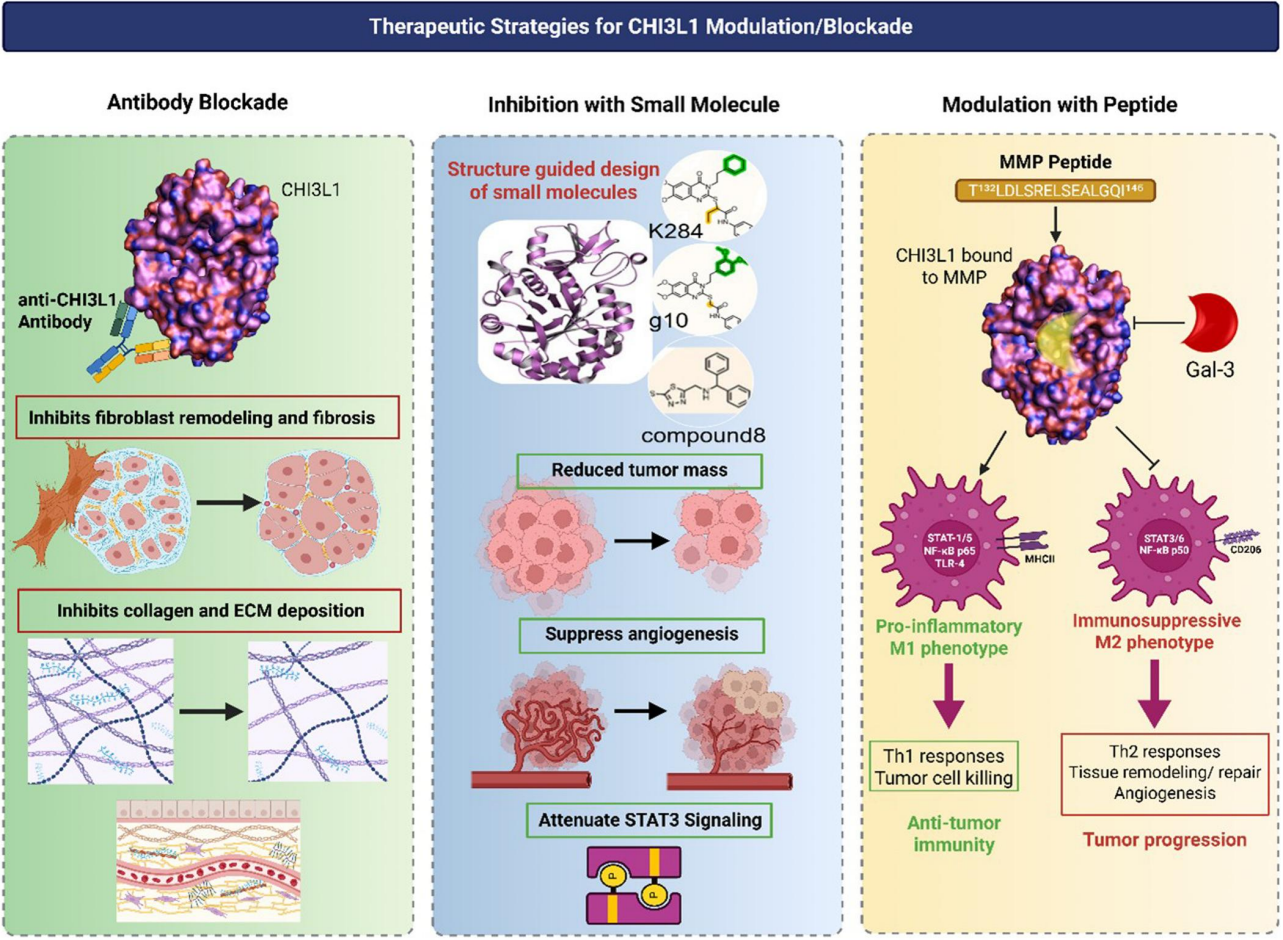
Therapeutic strategies for CHI3L1 modulation and blockade. (Left) Antibody blockade: Neutralizing anti‐CHI3L1 antibodies bind CHI3L1 and inhibit its downstream effects on fibroblast activation, extracellular‐matrix (ECM) remodeling, and collagen deposition, thereby reducing fibrosis. (Center) Small‐molecule inhibition: Structure‐guided design has yielded candidate inhibitors such as K284, g10, and compound 8 that target CHI3L1's pseudoactive groove. These small molecules reduce tumor mass, suppress angiogenesis, and attenuate STAT3 signaling. (Right) Peptide‐based modulation: A Galectin‐3–binding protein mimetic peptide (MMP; T^132^LDLSRELSEALGQI^146^) disrupts the CHI3L1–Galectin‐3 interaction, shifting macrophage polarization from an immunosuppressive M2 phenotype toward a pro‐inflammatory M1 phenotype. This reprogramming enhances Th1‐mediated antitumor immunity and limits tissue‐remodeling and angiogenic programs that support tumor progression. [Color figure can be viewed at wileyonlinelibrary.com]

Building on these foundations, combination strategies offer the most rational translational path. In IPF, CHI3L1 is upregulated in epithelial and stromal compartments and correlates with disease severity [124]. Antibody blockade reduces ECM accumulation and fibroblast activation in preclinical models, whereas pirfenidone and nintedanib act downstream on fibrotic signaling [125]. Dual inhibition by blocking both the initiator (CHI3L1) and the amplifier (fibrotic feedback loops) could achieve deeper tissue normalization than either alone. A similar strategy applies in cancer, where fibrotic stroma and aberrant vasculature restrict immune and drug access. CHI3L1 intersects with angiogenic control by stabilizing HIF1α and enhancing VEGF expression; in glioblastoma, its neutralization normalized tumor vasculature and improved chemotherapeutic delivery [23]. Combination with VEGF inhibitors such as bevacizumab could therefore achieve more complete vascular normalization, improving perfusion and immune infiltration [126]. Early data from pancreatic‐cancer models support this principle, demonstrating enhanced efficacy in the context of dense stroma and hypoxic vasculature.

Safety considerations are integral to translation. Because CHI3L1 contributes to wound healing, epithelial restitution, and vascular remodeling, systemic blockade could delay recovery from injury or surgery [73]. CHI3L1‐deficient mice indeed show delayed cutaneous wound closure, highlighting the need for controlled exposure. Local delivery strategies, such as inhaled siRNAs for pulmonary disease or intratumoral antibody injections in oncology can restrict systemic burden, while intermittent dosing cycles may permit transient suppression of pathogenic signaling without impairing physiological repair. Further selectivity might arise from exploiting CHI3L1's glycoform diversity: disease‐associated variants secreted by macrophages or fibroblasts differ in glycosylation from homeostatic epithelial forms, providing an opportunity for glycan‐directed antibodies or lectin‐mimetic drugs that spare protective isoforms [24, 127].

Small molecules offer a complementary angle. Fragment‐based screens have identified K284, which occupies CHI3L1's pseudoactive groove [117]. In metastatic melanoma models, K284 reduced tumor dissemination to the lung and liver, suggesting that the pseudoactive site, long dismissed as vestigial, can be pharmacologically exploited. Subsequent optimization of this scaffold through structure‐guided design, molecular docking, and biophysical validation (MST and SPR) yielded compound **11g**, a next‐generation CHI3L1 inhibitor with superior binding affinity and pharmacokinetic stability [44]. Compound **11g** displayed extended plasma half‐life (t_1_/_2_ ≈ 2–2.5 h in human and mouse), improved microsomal stability, and reduced intrinsic clearance, along with lower cytotoxicity toward astrocytes. In 3D glioblastoma (GBM) spheroids, **11g** reduced spheroid mass by over 50% and curtailed migration by ~60%, demonstrating its translational potential as a CNS‐active CHI3L1 inhibitor (Figure 7, center panel). Parallel structure‐based pharmacophore screening of > 4 million compounds identified alternative scaffolds beyond the K284 lineage; among these, compound 8 bound CHI3L1 with micromolar affinity, suppressed phospho‐STAT3 signaling, and inhibited angiogenesis in GBM spheroids, confirming that distinct chemotypes can effectively target CHI3L1's pseudoactive pocket (Figure 7, center panel) [44, 117, 128].

In addition to small‐molecule ligands, peptide‐based strategies have been explored to inhibit CHI3L1 function. A Galectin‐3–binding protein (Gal3BP) mimetic peptide (GMP; T^132^LDLSRELSEALGQI^146^) was designed to disrupt the CHI3L1–Galectin‐3 complex that mediates macrophage‐driven immune suppression in glioma (Figure 7, right panel) [48]. Molecular dynamics simulations confirmed that GMP mimics the Gal3BP interface and competitively prevents Gal3 binding to CHI3L1. Functionally, GMP inhibited CHI3L1‐induced M2 macrophage migration and reduced CHI3L1–Gal3 complex formation in THP‐1 cells, thereby attenuating macrophage‐mediated tumor progression and establishing GMP as a Gal3BP‐mimetic peptide capable of selectively disrupting CHI3L1–Gal3 signaling. RNA‐based modalities expand the toolkit further. siRNA knockdown of CHI3L1 in murine lung fibrosis models significantly decreased fibroblast activation and improved lung compliance [73]. Inhaled formulations of antisense oligonucleotides (ASOs) targeting CHI3L1 transcripts reduced airway remodeling in asthma models, offering a plausible path for localized delivery [129]. Together, these studies demonstrate that CHI3L1 can be directly neutralized at the protein level, intercepted at its receptor interface, or silenced at the transcript level, making it unusually versatile compared to other checkpoints.

These findings outline a practical translational framework in which CHI3L1 inhibition functions as a feasible and clinically relevant therapeutic strategy. Its activity across multiple intervention modalities, compatibility with immune‐checkpoint inhibitors, anti‐fibrotic and anti‐angiogenic agents, favorable safety characteristics with targeted delivery, and alignment with biomarker‐based patient stratification support its integration into combination treatments. By disrupting this non‐canonical suppressive pathway, CHI3L1‐directed therapies can enhance responses in refractory tumors, reduce pathological fibrosis, and modulate immune dysfunction in infectious and neuroinflammatory settings. Overall, durable therapeutic efficacy will likely require concurrent targeting of CHI3L1 in addition to established checkpoints such as PD‐1 and CTLA‐4.

## 10 Clinical Translation, Trials, and Future Perspectives

Translating CHI3L1 from a candidate checkpoint into a therapeutic axis now requires a deeper understanding of its biological context for precision targeting. As a secreted, extracellular pseudoenzyme present in plasma, stroma, and multiple cell types, CHI3L1 is both accessible to drugs and biologically complex. Blocking CHI3L1 requires distinguishing its role in pathological persistence from its part in normal tissue repair. Functionally, CHI3L1 shapes immune suppression, fibrosis, and angiogenesis through multi‐receptor hubs such as the IL‐13Rα2, TMEM219, and Galectin‐3 triad, acting less like a classical checkpoint at the immune synapse and more like a conductor that remodels the tissue microenvironment. This distinctive biology opens up exciting therapeutic possibilities but also raises critical questions about target selectivity, timing, and tissue‐specific effects.

Preclinical studies highlight the range of strategies for tackling CHI3L1. Neutralizing antibodies that interrupt IL‐13Rα2–TMEM219 interactions can reverse fibrosis and neovascularization. Small molecules, like fragment‐based inhibitor K284, g10 and compound 8 can engage its pseudoenzymatic cleft to suppress metastasis. RNA silencing or soluble receptor decoys can dampen upstream signaling. Together, these approaches show that CHI3L1 is “multiplex‐druggable” responsive to antibodies, small molecules, peptides, and nucleic acid‐based interventions. The challenge now is not whether we can target CHI3L1, but whether we can do so precisely: identifying the right disease contexts, immune states, and patient populations where intervention will deliver lasting benefit.

Growing evidence supports CHI3L1 blockade as a complementary approach to established immunotherapies. In solid tumors, CHI3L1 overexpression correlates with PD‐L1 upregulation and poor response to PD‐1 blockade [17, 130]. Experimental inhibition of CHI3L1 reprograms TAMs and restores cytotoxic T‐cell infiltration, sensitizing resistant tumors to immune checkpoint inhibitors [89]. In lung and colorectal cancer models, anti‐CHI3L1 antibodies reduce tumor vascularization and synergize with anti‐VEGF or anti‐PD‐1 therapy to improve local immune activation and treatment efficacy [49, 90]. These studies collectively position CHI3L1 as a stromal and metabolic “barrier node” whose inhibition enhances the efficacy of canonical checkpoint blockade.

Building upon these preclinical findings, the translational logic extends naturally into broader disease contexts and trial design frameworks. Combination therapies offer the clearest near‐term opportunities. In oncology, CHI3L1 blockade could help “cold,” PD‐1 resistant tumors by breaking down stromal and metabolic barriers that block T‐cell infiltration. In fibrosis, pairing CHI3L1 inhibitors with antifibrotic drugs may more effectively suppress fibroblast activation and matrix deposition. In neuroinflammation, combining them with microglial modulators could restore glial balance and limit maladaptive tissue remodeling. Adaptive trial designs similar to those that established PD‐1 and LAG‐3 combinations could test CHI3L1 targeting therapies as monotherapies or bispecific antibodies that integrates PD‐1 inhibition.

Unanswered questions now focus on precision, biomarkers, and longitudinal safety. Plasma CHI3L1 levels correlate with disease severity in cancer, fibrosis, and neuroinflammatory disorders, but whether these levels predict therapeutic responsiveness remains unclear [57]. Multi‐omics studies suggest CHI3L1 expression accompanies a myeloid‐dominant immune state, providing a potential signature for patient stratification [131]. Another key question is the role of paralogs such as CHI3L2 and acidic mammalian chitinase (CHIA), which may compensate following CHI3L1 inhibition [132, 133]. Addressing these gaps will require tissue‐resolved, time‐course analyses and integration of proteomic and spatial transcriptomic data from early‐phase trials.

Clinical validation will depend on demonstrating targeting CHI3L1 as a checkpoint reversal rather than mere biomarker decline, This includes demonstrable restoration of CD28/ICOS costimulation and cytokine production within tumors, induction of fibroblast quiescence with improved tissue compliance in fibrotic disease, and attenuation of neuroinflammatory signatures across CNS pathologies [134]. Equally important is early and rigorous safety classification, as systemic CHI3L1 inhibition could impair wound healing and angiogenesis, making precision strategies such as glycoform‐selective antibodies, localized delivery platforms, and intermittent dosing schedules, essential to preserve physiological repair mechanisms. If these challenges are met with the same mechanistic precision that shaped earlier immune checkpoint therapies, CHI3L1 blockade has the potential to unify seemingly unrelated pathological states like cancer, chronic fibrosis, persistent infection, and neurodegeneration, under a shared therapeutic strategy. By dismantling extracellular pseudoenzymatic scaffolds that elevate the activation threshold of immune and stromal systems, CHI3L1 targeting reframes checkpoint biology beyond the confines of the immune synapse and into the structural, metabolic, and reparative architecture of disease. In doing so, it positions CHI3L1 as a compelling next‐generation axis in translational immunotherapy.

## Author Contributions

**Kirti Upmanyu:** conceptualization, methodology, software, data curation, formal analysis, investigation, validation, visualization, writing – original draft, writing – review and editing. **Saurabh Upadhyay:** data curation, formal analysis, investigation, validation, visualization, writing – original draft, writing – review and editing. **Moustafa T. Gabr:** conceptualization, resources, project administration, funding acquisition, supervision, writing – review and editing.

## Conflicts of Interest

The authors declare no conflicts of interest.

## Data Availability

Data sharing not applicable to this article as no datasets were generated or analysed during the current study.
